# Engineering Versatile Bacteria‐Derived Outer Membrane Vesicles: An Adaptable Platform for Advancing Cancer Immunotherapy

**DOI:** 10.1002/advs.202400049

**Published:** 2024-07-01

**Authors:** Ziheng Luo, Xiang Cheng, Bin Feng, Duoyang Fan, Xiaohui Liu, Ruyan Xie, Ting Luo, Seraphine V. Wegner, Dayou Ma, Fei Chen, Wenbin Zeng

**Affiliations:** ^1^ Xiangya School of Pharmaceutical Sciences Central South University Changsha 410013 China; ^2^ Hunan Key Laboratory of Diagnostic and Therapeutic Drug Research for Chronic Diseases Changsha 410078 China; ^3^ Institute of Physiological Chemistry and Pathobiochemistry University of Münster 48149 Münster Germany

**Keywords:** bacterial engineering, cancer immunotherapy, hybrid membrane vesicles, outer membrane vesicles, surface modification

## Abstract

In recent years, cancer immunotherapy has undergone a transformative shift toward personalized and targeted therapeutic strategies. Bacteria‐derived outer membrane vesicles (OMVs) have emerged as a promising and adaptable platform for cancer immunotherapy due to their unique properties, including natural immunogenicity and the ability to be engineered for specific therapeutic purposes. In this review, a comprehensive overview is provided of state‐of‐the‐art techniques and methodologies employed in the engineering of versatile OMVs for cancer immunotherapy. Beginning by exploring the biogenesis and composition of OMVs, unveiling their intrinsic immunogenic properties for therapeutic appeal. Subsequently, innovative approaches employed to engineer OMVs are delved into, ranging from the genetic engineering of parent bacteria to the incorporation of functional molecules. The importance of rational design strategies is highlighted to enhance the immunogenicity and specificity of OMVs, allowing tailoring for diverse cancer types. Furthermore, insights into clinical studies and potential challenges utilizing OMVs as cancer vaccines or adjuvants are also provided, offering a comprehensive assessment of the current landscape and future prospects. Overall, this review provides valuable insights for researchers involved in the rapidly evolving field of cancer immunotherapy, offering a roadmap for harnessing the full potential of OMVs as a versatile and adaptable platform for cancer treatment.

## Graphical abstract

Outer membrane vesicles (OMVs) derived from bacteria present an adaptable platform for cancer immunotherapy. The unique properties of OMVs underscore their potential, including natural immunogenicity and accessibility to engineering for specific therapeutic goals. They exhibit excellent biocompatibility, drug‐loading capacity, and modifiability, which enable modifications through genetic engineering, surface functionalization, the construction of hybrid membrane vesicles and drug delivery platforms.

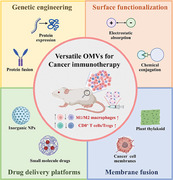

## 1 Introduction

Cancer stands as the primary contributor to mortality in developed nations and ranks second in terms of fatalities in developing regions.^[^ ^1^, ^2^ ^]^ In 2020, the International Agency for Research on Cancer (IARC), a division of the World Health Organization (WHO), reported a notable rise in new cancer cases, totaling 19.3 million worldwide. Tragically, this surge in cancer incidence was accompanied by a staggering 10 million cancer‐related deaths globally.^[^ ^3^ ^]^ While early chemotherapy has proven effective in achieving rapid tumor remission, a significant challenge arises with the development of resistance in tumor cells during prolonged treatment. This resistance often leads to metastasis and recurrence, ultimately resulting in treatment failure and mortality. To tackle the issue of resistance to a single chemotherapeutic agent, a two‐pronged approach has been taken. First, combination therapy has been embraced, leading to increasingly intricate treatment regimens. Second, different dosage intensities, including shorter intervals or higher doses of chemotherapy, have been explored. Despite these efforts, progress in chemotherapy success has plateaued. Traditional treatments such as surgery, radiotherapy, and polychemotherapy have demonstrated limited efficacy in various tumor types. To break this impasse, the introduction of innovative therapeutic modalities like immunotherapy marks a significant advancement. Immunotherapy offers a crucial leap forward in addressing the challenges posed by resistance and provides a promising avenue for more effective cancer treatment.

Immunotherapy, a pivotal approach in cancer treatment, capitalizes on the body's own immune system.^[^ ^4^ ^]^ This strategy hinges on the recognition of tumor antigens on cancer cell surfaces, forming the basis of cancer immunity. Immunotherapy stands out for its superior efficacy in curtailing tumor metastasis and preventing recurrence compared to alternative treatments.^[^ ^5^ ^]^ Currently, prominent immunotherapeutic avenues encompass tumor vaccines,^[^ ^6^ ^]^ chimeric antigen receptor T‐cell (CAR‐T) therapy,^[^ ^7^ ^]^ and immune‐checkpoint blockade (ICB).^[^ ^8^ ^]^ These approaches have demonstrated impressive efficacy across various cancers. However, CAR‐T cell therapy encounters formidable challenges in treating solid tumors, including issues like antigen escape, off‐target effects, immunosuppression within the tumor microenvironment (TME), and limited tumor infiltration.^[^ ^9^ ^]^ Similarly, ICB therapies exhibit efficacy in only a small subset of patients, with a positive response observed in merely 10–30% of cases.^[^ ^10^, ^11^ ^]^ Despite the success of preventive vaccines such as the human papillomavirus (HPV) vaccines, progress in individualized therapeutic vaccines has been sluggish. The first therapeutic anti‐prostate cancer vaccine Provenge emerged in 2010, but the overall success rate in oncology vaccine development remains below 1%, with several clinical phase III programs ending in failure. Concurrently, immune suppression induced by irregular metabolism, hypoxia, and acidic conditions within the TME significantly hinders treatment outcomes.^[^ ^12^, ^13^ ^]^ Given these challenges, there is an imperative to explore innovative avenues in immunotherapy to surmount the obstacles associated with conventional approaches to treating solid tumors.

Bacterial extracellular vesicles (BEVs) are a diverse category of membrane‐associated substances produced by cultivated bacteria and exhibit considerable heterogeneity in terms of their origin, size, composition, and functions. These vesicular entities are commonly denoted as membrane vesicles, microvesicles, or exosomes. In terms of classification of membrane vesicles, bacterial vesicles secreted by Gram‐negative bacteria are commonly named outer membrane vesicles (OMVs). In retrospect, OMVs have been studied for more than fifty years and were originally discovered in *Escherichia coli* (*E. coli*) in 1965.^[^ ^14^ ^]^ After the first observation of these vesicles secreted by bacterial out membrane using electron microscopy,^[^ ^15^ ^]^ a growing number of studies have confirmed the existence of OMVs. In the 1980s, with the development of electron microscopy techniques, researchers devoted to the structural characterization and composition identification of OMVs, which were found to contain lipids, proteins, and other biomolecules from the bacterial outer membrane. Owing to their bacterial origin, the immunological properties of OMVs became a subject of interest in the 1990s. It's evidenced that OMVs are bilayer vesicles of spherical nanoparticles (20‐200 nm) that consist of the outer leaflet of lipopolysaccharides (LPS) and other pathogen‐associated molecular patterns (PAMPs), and the inner leaflet of peptidoglycan and periplasmic proteins.^[^ ^16^ ^]^ As part of the cargo transporter, OMVs are involved in gene transfer, stress relief, and cellular recognition, implying that they play an important role in bacterial activities such as cell communication, biofilm formation, and pathogenesis.^[^ ^17^ ^]^

**Figure 1:**
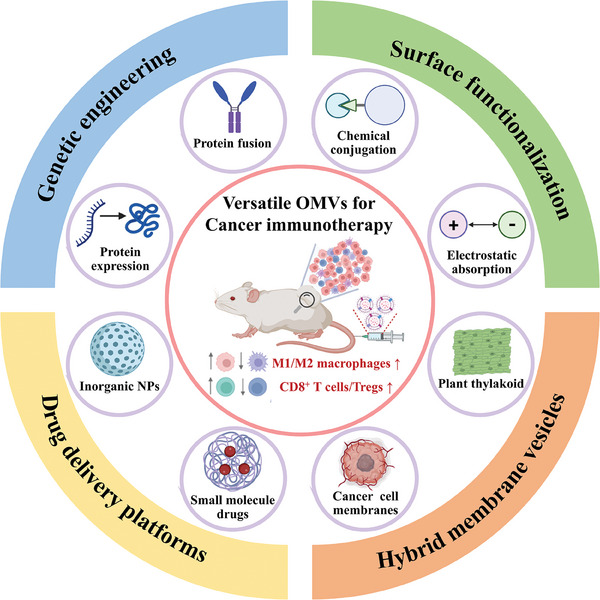
Overview of versatile modified OMVs for cancer immunotherapy. There are four strategies for OMVs tailoring including genetic engineering by protein expression and fusion, surface functionalization by chemical conjugation and electrostatic absorption, drug delivery platforms by loading inorganic nanoparticles and small molecule drugs, hybrid membrane vesicles by fusing with cancer cell membranes and plant thylakoid.

In earlier research, OMVs were initially perceived as by‐products of bacterial growth. However, recent studies over the past decade have unveiled a significant role for bacterial‐secreted OMVs in modulating host immune responses. This is attributed to the abundance of PAMPs on their surfaces. OMVs, characterized by nanoscale vesicle structures comprising phospholipids and proteins, exhibit active recognition and attraction by antigen‐presenting cells, thereby effectively triggering immune responses. Moreover, owing to their bioproperties such as excellent biocompatibility, superior drug‐loading capacity, and versatility in modification, OMVs have emerged as crucial players in cancer immunotherapy. **Figure** **1** illustrates their diverse functionalization through genetic engineering, surface functionalization, drug delivery platforms, and hybrid membrane nanovesicles. This multifaceted approach underscores the growing significance of OMVs in the realm of cancer immunotherapy. In recognition of the significant potential within this emerging field, this review seeks to consolidate the diverse roles played by different extracellular vesicles, particularly OMVs within the TME. The objective is to examine recent advancements and their practical implications in the fight against cancer. It is our conviction that the comprehension of the clinical possibilities of OMVs is only in its infancy, and further investigation into this avenue of research holds promise for more efficacious cancer therapies. Of paramount importance, this review offers discerning insights and outlines prospective pathways for addressing the existing limitations and challenges pertaining to safety, manufacturing, and techniques. By mitigating redundancy, we aim to streamline the discussion and accentuate the unique opportunities that the exploration of OMVs presents for enhancing the landscape of cancer treatment.

## 2 Biogenesis and Preparation of OMVs

**Figure 2:**
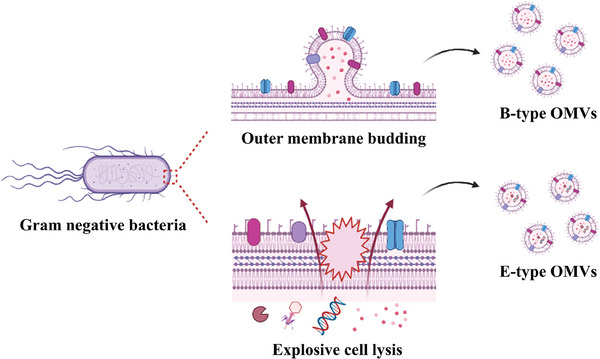
Schematic illustration of different production mechanisms of B‐type OMVs and E‐type OMVs secreted from Gram‐negative bacteria. The blebbing of the outer membrane of living bacterial cells generates B‐type OMVs, while membrane disintegration during explosive cell lysis yields E‐type OMVs.

Based on studies to date, the approaches of generation involved in OMVs can be divided into two dominant categories. One is the generation of OMVs by the blebbing and subsequent detaching of the extracellular outer membranes (OM) of living cells during certain bacterial activity.^[^ ^18^ ^]^ The other is the loss of membrane integrity and explosive cell lysis, which then leads to cell death.^[^ ^19^ ^]^ Based on their differences in formation, they can be categorized into budding‐type OMVs (B‐type OMVs) and explosive cell lysis‐type OMVs (E‐type OMVs), as schematically depicted in **Figure** **2**. In proposing how B‐type OMVs vesiculation occurs, there are three primary hypotheses:

1. Within the hypothesis of membrane cross‐linking regulation,^[^ ^20^ ^]^ lipoprotein (LPP) resided in the periplasm of Gram‐negative bacteria established a linkage between the outer membrane region and the peptidoglycan layer. When LPP undergoes deconstruction, the linkage between the two layers weakens or vanishes. Consequently, the outer membrane may become protruding, ultimately resulting in the production and release of OMVs.^[^ ^21^ ^]^
2. The pressure hypothesis, also known as cell stress hypothesis, suggests that within bacterial periplasmic space, numerous segments of peptidoglycan and misfolded proteins are present.^[^ ^22^ ^]^ Typically, these substances accumulate, aggregate, and exert pressure on the plasma membrane, causing a gradual separation of the outer membrane from the peptidoglycan layer, leading to the production and subsequent release of OMVs.^[^ ^23^ ^]^ Bacteria can employ this mechanism to excrete redundant or potentially harmful substances, effectively alleviating the pressure and promoting their survival.
3. The lipid enrichment hypothesis pertains to the composition of the cell membrane, primarily comprising lipids and proteins. The outer membrane budding is resulted from cell envelope chaos, including disturbed peptidoglycan biosynthesis, accumulation of misfolded proteins, and inserting of hydrophobic molecules into the outer membrane. Thus, the cargo contents of B‐type OMVs mainly comprised of periplasmic proteins and outer membrane constituents, such as LPS, denatured proteins, and peptidoglycan. It's worth mentioning that this type of OMVs is devoid of cytosolic components such as DNA and RNA, indicating its biosafety and biocompatibility to a certain extent.^[^ ^24^ ^]^

In addition to vesicles originating from live bacteria, OMVs have also been discovered in some dead bacteria.^[^ ^25^ ^]^ Another type of OMVs secretion mechanism is accompanied by membrane disintegration during explosive cell lysis, namely E‐type OMVs.^[^ ^26^ ^]^ In 2016, Cynthia B. et al.^[^ ^27^ ^]^ revealed that explosive cell lysis was a mechanism for the production of OMVs. Under the stress of genotoxicity, explosive cell lysis is triggered to activate the expression of endolysins, which originate from natural microbe fighter phages. The bacterial peptidoglycan layer is degraded from the inside by endolysins and also disintegrated by endolysins released by neighboring bacteria. Once the holes are formed on the surface of peptidoglycan, the cytoplasmic membrane is extruded and fragmented through the holes. Due to the high cell turgor, the final membrane fragments are aggregated and self‐assembled to form E‐type OMVs. According to the mechanism of formation, this type of OMVs is richer in cargo contents such as cytosolic substances, DNA or RNA genetic material, phages, and endolysins than that of B‐type OMVs.

Achieving a harmonious equilibrium between the immunogenicity and biocompatibility of isolated OMVs is crucial to ensuring ample safety for subsequent research endeavors. Hence, various prevalent physical methods have emerged for OMVs extraction to address this requirement. The prevailing extraction techniques encompass a spectrum of approaches such as differential centrifugation (DC), density gradient centrifugation (DGC), ultrafiltration (UF), precipitation, as well as immunoaffinity and size‐exclusion chromatography (SEC).^[^ ^28^ ^]^ The various isolation approaches possess distinct mechanisms, advantages, disadvantages, and performance properties, which are succinctly summarized in **Table** **1**. To achieve higher isolation efficiency, a combination of these techniques, such as ultracentrifugation‐ultrafiltration, has been most commonly used. Protocols for the preparation of OMVs have been fully studied previously.^[^ ^29^ ^]^ Briefly, the parent bacteria are removed from a pre‐incubated bacteria culture medium with low‐speed oscillation. The resulting liquid is then concentrated by ultrafiltration to remove residual debris and bacteria. To collect the purified OMVs, the concentrated supernatant is subjected to ultracentrifugation.

**Table 1:** Various evaluation parameters of different isolation approaches to isolate OMVs, along with their mechanisms, advantages, and disadvantages.

	DC	DGC	UF	Precipitation	Immunoaffinity	SEC
Mechanism	separation based on size, shape, and density through sequential rounds of centrifugation	separation based on buoyant density	separation based on molecular weight cutoff	separation based on sedimentation between target substances and precipitant	Separation based on interactions between antibody and surface protein	separation based on size
Advantages	high yield and simple operation	high purity and yield	high efficiency and fast process	low costs and use in large‐scale isolation	high purity and yield	high purity and good biocompatibility
Disadvantages	high instrument requirement and low purity	high instrument requirement and time consuming	product damage and sample loss	complicated operation and time‐consuming	high cost and only use in small‐scale isolation	complicated operation and time consuming
Scale	High	Low	Medium	High	Low	Low
Efficiency	Medium	Low	Medium	High	Medium	Medium
Purity	Low	High	Medium	Low	High	High
Operability	High	Low	High	High	Low	Low
Yield	Medium	Medium	High	High	High	Medium
References	[30]	[31]	[32]	[33]	[34]	[35]

## 3 Roles of OMVs in Tumor Microenvironment

### 3.1 Characterization of Immunosuppressive Tumor Microenvironment

The intricate TME arises from dynamic interplay of various cellular and non‐cellular elements. Rather than a solitary actor, it functions as a collective ensemble comprising cancer cells, fibroblasts, endothelial cells, and immune cells.^[^ ^36^ ^]^ This intricate assembly is the outcome of host innate response, which includes cytokines, chemokines, and inflammation, responses that disrupt cancer behavior, tumor surface antigens, cellular defense and growth.^[^ ^37^ ^]^ Numerous studies have solidified the role of TME in forecasting patient prognosis and predicting treatment responses. A diverse array of immune cells populates the TME, encompassing lymphocytic cells (T cells, B cells, and natural killer cells) and myeloid cells (neutrophils, macrophages, and dendritic cells). Furthermore, a variety of effector and regulatory cells involved in adaptive immunity are present in the TME, including Type 1 T helper cells (Th1), Type 2 T helper cells (Th2), and T regulatory cells (Tregs).^[^ ^38^ ^]^ Among these immune actors, infiltrating lymphocytes and dendritic cells (DCs) play a beneficial prognostic role in prognosis, while M2 macrophages and polymorphonuclear cells are categorized as less favorable cellular components.

Based on the composition of the immune infiltrate, tumors can be categorized as “immune hot” and “immune cold”. Immune hot tumors are characterized by elevated levels of programmed death ligand‐1 (PD‐L1) expression and an abundance of cytotoxic T lymphocytes (CTLs) expressing programmed death 1 (PD‐1).^[^ ^39^ ^]^ To exclude autoimmunity, the immune system has evolved a variety of “preventive mechanisms”, which are expressed in the form of immune checkpoints to reduce the probability of autoimmunity and protect normal tissues from damage during immune activation. PD‐1 and PD‐L1 are a typical pair of immune checkpoints, and their interaction can regulate the immune response. Within immune hot tumors, the infiltrating lymphoid and myeloid cells exhibit distinctive capabilities. For instance, monocytes that have undergone M1 polarization release lysosomes, which play a role in eradicating tumor cells. Additionally, natural killer (NK) cells possess the ability to lyse cells with reduced major histocompatibility complex (MHC) protein expression, including cancer cells that have undergone mutations.^[^ ^40^ ^]^ Immune cold tumors, often referred to as non‐inflamed tumors, are characterized by the absence of tumor cells expressing PD‐L1 and CTLs expressing PD‐1. There are multiple factors that contribute to the immune cold phenotype in tumors. These include diminished antigenic expression,^[^ ^41^ ^]^ adoption of effective immune evasion by the tumor, rejection of T cells from vasculature, and heightened levels of immunosuppressive immune infiltration. Notably, immune cold tumors exhibit elevated levels of myeloid‐derived stromal cells (MDSCs), M2 macrophages, Tregs, and Th17 cells.^[^ ^42^, ^43^ ^]^ Among these components, M2‐polarized macrophages and Tregs play pivotal roles in promoting angiogenesis by releasing adrenomedullin and vascular endothelial growth factors (VEGFs).^[^ ^44^ ^]^ Additionally, immunosuppressive components such as interleukin‐10 (IL‐10), PD‐L1, and transforming growth factor‐β (TGF‐β) were overexpressed, which contribute to a favorable environment for tumor growth. In essence, the immune system's role in regulating tumorigenesis and progression is paramount, and it also serves as a predictor of therapeutic efficacy throughout the intricate process of tumorigenesis. However, tumors possess inherent mechanisms to evade immune surveillance and establish a conducive milieu for their survival and proliferation. Therefore, it's significantly urgent to explore a way to modulate the TME, activate immune cells, thus restoring normal immune systems. In this context, OMVs are a good candidate.

### 3.2 Tumor Targeting Mechanism of OMVs

The current viewpoint indicates that tumor cells need more nutrients and oxygen for rapid growth, and therefore secrete VEGFs and other growth factors related to tumor angiogenesis. The newly generated tumor blood vessels are very different from normal blood vessels in structure and morphology, such as the large endothelial cell gaps, absence of the smooth muscle layer of the vessel wall, and deficiency of angiotensin receptor function. In addition, tumor tissues also lack lymphatic vessels, resulting in the obstruction of lymphatic fluid return.^[^ ^45^ ^]^ These structural changes result in the phenomenon that large molecules can easily cross the vascular walls and accumulate at the tumor sites for a long period, thus avoiding clearance by lymphatic drainage. Therefore, this is known as the “enhanced permeability and retention effect” (EPR) in solid tumors, which allows passive targeting of nanosized OMVs.^[^ ^46^ ^]^ According to the kinetic studies, OMVs enter blood vessels via body circulation after administration, penetrate the tumor vasculature and remain at the tumor site through the EPR effect, which implies the potential of drug‐loaded OMVs as a nanoplatform for tumor‐targeting.^[^ ^47^ ^]^

Hypoxia microenvironment is a widespread hallmark in nearly 90% of solid tumors.^[^ ^48^ ^]^ Hypoxia is closely related to tumor proliferation, differentiation, angiogenesis, and energy metabolism, as well as the occurrence of cancer drug resistance and poorer patient prognosis, and is the culprit behind the constant ravaging of cancer cells.^[^ ^49^ ^]^ Fortunately, some living bacteria naturally exhibit tumor tropism due to their preference for the hypoxic environment in which tumor cells exist,^[^ ^50^ ^]^ and thus bacterially secreted OMVs demonstrate the potential ability to actively localize tumor tissue. Meanwhile, a growing number of studies have identified that bacteria, as an important part of TME, have a tendency to colonize under hypoxic conditions within tumors and play a prominent role in driving tumor metastasis.^[^ ^51^ ^]^ Therefore, OMVs secreted by these parent bacteria, when used for tumor therapy, can specifically recognize intratumor microbes through homing effect, and are also biocompatible and do not cause harm to other healthy tissues in body. Overall, OMVs show promising natural tropism for malignant tumors, both in passive and active targeting.

### 3.3 Immune Responses Elicited by OMVs

In addition to their advantages in tumor targeting, OMVs have greater scope in the field of regulating the immune microenvironment. A large number of studies have evidenced that OMVs can be detected by innate immune cells via the activation of surface toll‐like receptors (TLRs), which results in the production of pro‐inflammatory cytokines and chemokines.^[^ ^52^ ^]^ Specifically, as key components of immunogenicity, PAMPs can be delivered by OMVs, and recognized by pattern recognition receptors (PRRs) like TLRs of immune cells, thereby triggering cytokine production, inflammation and programmed cell death. Moreover, different species of Gram‐negative bacteria can mediate immune responses through different TLRs, and the main inflammatory factor changes are summarized in **Table** **2**.

**Table 2:** Summary of different TLRs‐mediated immune responses.

Bacteria species	Types of TLRs	Inflammatory factor change	References
*E. coli*	TLR4,TLR5	IL‐6, IL‐8, IL‐1β,TNF‐α	[53]
*Pseudomonas aeruginosa* (*P. aeruginosa*)	TLR4	IL‐6, IL‐8, IL‐1β,TNF‐α	[54]
*Klebsiella pneumonia* (*K. pneumonia*)	TLR2, TLR4	IL‐8	[55]
*Salmonella typhimurium* (*S. typhimurium*)	TLR2, TLR4, TLR5, TLR9	IL‐12, TNF‐α	[56, 57]
*Porphyromonas gingivalis* (*P. gingivalis*)	TLR2, TLR4,TLR7, TLR8, TLR9	IL‐6, IL‐8, IL‐1β,TNF‐α	[58]
*Helicobacter pylori* (*H. pylori*)	TLR2,TLR4,TLR10	IL‐8, IL‐6, IL‐1β	[59, 60]
*Moraxella catarrhalis* (*M. catarrhalis*)	TLR2	IL‐8	[61]
*Acinetobacter baumannii* (*A. baumannii*)	TLR2, TLR4	IL‐6	[62]

Due to the TME, DCs are in an immature state and cannot effectively recognize the inflammatory environment, resulting in immune inactivation. Upon administration of OMVs, the PAMPs on the surface of OMVs are able to interact with the TLR4 on immature DCs to promote their maturation. In this process, DCs possess the capability to metabolize antigens and subsequently display antigenic epitopes on their exterior, and employ the MHC complex as a presentation vehicle. Following this modification, the transformed antigen can be detected by the T cell antigen receptor situated on the surface of T cells, which activates T cells and triggers an immune response that engages both CTLs and helper T cells.^[^ ^63^ ^]^ Specifically, DCs serve as pivotal components in orchestrating the immune response, effectively connecting the innate and adaptive immune systems.^[^ ^64^, ^65^ ^]^ Researches have demonstrated that OMVs possess the capability to enter DCs, leading to the upregulation of CD86 and MHC‐II molecules on the surface of DCs. This internalization also triggers the production of cytokines like tumor necrosis factor‐α (TNF‐α) and IL‐12.^[^ ^66^ ^]^

Tumor‐associated macrophages (TAMs), among the most abundant leukocytes infiltrating various tumor types, undergo distinct education within the TME, resulting in diverse phenotypes.^[^ ^67^ ^]^ M1‐like TAMs, often referred to as classically activated macrophages, exhibit anti‐cancer properties by releasing nitric oxide (NO) and inciting naive T cells to generate a Th1/cytotoxic response.^[^ ^68^ ^]^ In contrast, the tumor milieu predominantly houses M2‐like TAMs, termed alternatively activated macrophages, which actively foster tumor proliferation, angiogenesis, metastasis, and immune evasion.^[^ ^69^ ^]^ M2‐like TAMs employ various mechanisms to directly or indirectly suppress T cell functions, including the expression of immune checkpoint ligands for T cells like PD‐L1,^[^ ^70^ ^]^ the secretion of inhibitory cytokines such as IL‐10 and TGF‐β,^[^ ^71^ ^]^ and the recruitment of immunosuppressive cells like Tregs.^[^ ^72^ ^]^ Consequently, reprogramming M2‐like TAMs toward the M1 phenotype emerges as a promising approach to enhance T cell‐mediated anti‐tumor immunity and mitigate the immunosuppressive environment within tumors.^[^ ^73^, ^74^, ^75^ ^]^ Given the immunoadjuvant property, increasing studies have found that OMVs can regulate the immunosuppressive TME by repolarizing M2‐like TAMs into M1‐like phenotype. In addition, OMVs can also mediate PRRs‐independent host immunity to promote tumor treatment. Among the crucial cell types, macrophages are responsible for vigilant surveillance of host tissues, engaging in the phagocytosis of both bacteria and secreted OMVs. They possess the remarkable ability to swiftly recognize and counteract invading pathogens. Additionally, they release an array of products that serve as triggers for subsequent immune responses. Conversely, macrophages can act as host cells for specific pathogenic microorganisms.^[^ ^76^ ^]^ Researches have demonstrated that the internalization of OMVs into macrophages can trigger the production of pro‐inflammatory cytokines, involving various microbial sensors within the innate immune system. Notably, these sensors encompass nucleotide oligomerization domain‐like receptors (NLRs), including the NLR family member NLR‐pyrin domain containing 3 (NLRP3). Activation of NLRP3 by OMVs culminates in the assembly of an intracellular complex referred to as the inflammasome. This protein complex plays a pivotal role in facilitating the maturation of caspase‐1, subsequently leading to the secretion of mature cytokines from the IL‐1 family, including IL‐1β and IL‐18.^[^ ^77^ ^]^

**Figure 3:**
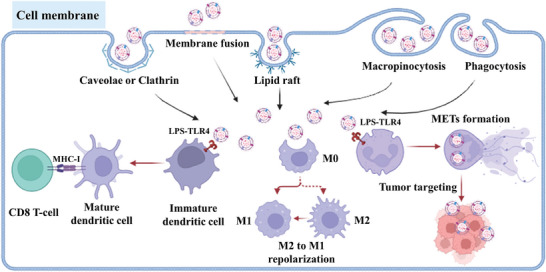
Schematic illustration of the approaches of OMVs enter host cells involves various pathways, such as caveolae or clathrin‐mediated endocytosis, membrane fusion, lipid rafts, macropinocytosis, and phagocytosis. The interactions with DCs, macrophages, and neutrophils elicit immune responses by promoting DCs maturation, M2‐to‐M1 repolarization, and NETs formation.

It is a widely acknowledged fact that early stages of tumor development are characterized by the presence of an inflammatory microenvironment. This environment recruits a considerable influx of immune cells that home in the tumor tissue through a phenomenon known as chemotaxis.^[^ ^78^, ^79^ ^]^ The migratory patterns of these immune cells can be harnessed as an innovative delivery approach for precise tumor‐targeted delivery. Among these immune cells, neutrophils are the most prevalent subtype and have gained extensive attention due to their remarkable ability to navigate through inflamed tissues.^[^ ^80^ ^]^ Owing to the features of PAMPs, OMVs can be recognized by neutrophils via TLR4, with delivery mediated by neutrophils.^[^ ^81^ ^]^ The interactions between OMVs and neutrophils can be classified into two distinct categories: immunomodulatory influences and effects related to the neutrophil extracellular traps (NETs).^[^ ^82^ ^]^ Neutrophils can indirectly respond to chemoattractants and chemokines, instigating their migration to the infection sites. These signaling molecules, like proinflammatory cytokine IL‐6, which is primarily released by macrophages, have the capacity to attract and summon neutrophils. In turn, neutrophils fulfill crucial roles in the effective elimination of infectious agents during acute infection.^[^ ^83^ ^]^ As shown in **Figure** **3**, when OMVs enter host cells via a range of mechanisms involving caveolae^[^ ^84^ ^]^ or clathrin‐mediated endocytosis,^[^ ^85^ ^]^ direct membrane fusion,^[^ ^86^ ^]^ indirect fusion with lipid rafts,^[^ ^87^ ^]^ macropinocytosis,^[^ ^88^ ^]^ and phagocytosis,^[^ ^89^ ^]^ the pathogenic features of PAMPs on the surface of OMVs promote their interactions with immune cells, such as immature DCs, macrophages, and neutrophils, finally inducing immune responses through different pathways.

## 4 Tailoring OMVs through a Versatile Engineering Strategy

### 4.1 Genetic Engineering of OMVs for Cancer Immunotherapy

Conventional cancer therapies often encounter challenges related to the indiscriminate distribution of therapeutic agents, which may result in detrimental side effects in healthy tissues. As one of the most promising technologies of the 21st century, bioengineering techniques offer innovative strategies and tools for the prevention, diagnosis, and treatment of cancer, which greatly enhance the targeting and efficacy of therapies in the field of cancer therapy. Briefly, the application of bioengineered technology to cancer therapy offers several advantages over traditional cancer therapies:

1. Bioengineered therapies can be designed to specifically target cancer cells, minimizing damage to healthy tissues. For example, engineered drug delivery systems can enhance drug solubility and reduce toxicity by delivering drugs directly to the tumor site.^[^ ^90^ ^]^
2. In response to the shortcomings and limitations of existing therapeutic techniques, genetic engineering technology can be used to selectively recombine genes and express relevant proteins to regulate tumor growth. Moreover, bioengineered therapies can be personalized based on a patient's unique genetic profile and tumor characteristics, increasing treatment effectiveness.^[^ ^91^ ^]^

On this basis, bioengineered OMVs have been explored as a novel platform for cancer therapy. Bioengineered OMVs encompass two approaches: OMVs produced by engineered bacteria and directly modified isolated OMVs. Compared to modifying OMVs directly by chemical or biological techniques, genetically engineered bacteria‐derived OMVs inherit relevant genetic segments, simplifying the preparation process, reducing production costs and most importantly, ensuring natural biocompatibility and bioactivity. Additionally, different from other platforms currently developed for adoptive cancer immunotherapy such as artificial antigen‐presenting cells (aAPCs),^[^ ^92^ ^]^ which are limited by manufacturing complexity, potential reduced immunogenicity, and challenges in ensuring uniform and stable antigen presentation, bioengineered OMVs inherit natural immunogenic properties to provide a versatile and biocompatible alternative. OMVs can carry multiple antigens and immunomodulatory agents, offering a more holistic approach to immune activation.

Modification of OMVs through bioengineered technology involves manipulating the genetic content of parent bacterial cells to influence the composition and properties of the resulting OMVs. For example:

1. Inserting genes encoding specific antigens or neoantigens relevant to the target cancer cells for antigen presentation. Utilizing the “Plug‐and‐Display” engineering technology that allows for the decoration of OMVs with various antigens,^[^ ^93^, ^94^ ^]^ Nie and Zhao's group made a major breakthrough in OMVs‐based mRNA vaccine delivery platform by fusing OMVs surface protein cytolysin A (ClyA) with RNA binding protein L7Ae and lysosomal escape protein listeriolysin O.^[^ ^95^ ^]^
2. Incorporating genes encoding ligands or antibodies on the surface of OMVs to enable specific targeting of cancer cells. Jon et al.^[^ ^96^ ^]^ selected human epidermal growth factor receptor 2 (HER2) as a tumor target and developed cancer‐specific targeting OMVs by expressing anti‐HER2 affibodies on the surface. This system can achieve specific accumulation in tumor tissue, thereby reducing cytotoxic effects.
3. Recombining genes for immunomodulatory molecules such as siRNA or miRNA that can stimulate immune responses. Jiang et al.^[^ ^97^ ^]^ reported an OMVs‐based drug delivery system, which was loaded with regulated in development and DNA damage response 1 (Redd1)‐siRNA. The delivery of Redd1‐siRNA can promote the downregulation of Redd1, thereby inhibiting tumor progression.

Thus, based on the different applications available for bioengineered OMVs, they can be classified into three categories: immunologic adjuvant, immunotherapeutic agent, and tumor vaccine.

#### 4.1.1 Immunologic Adjuvant

**Figure 4:**
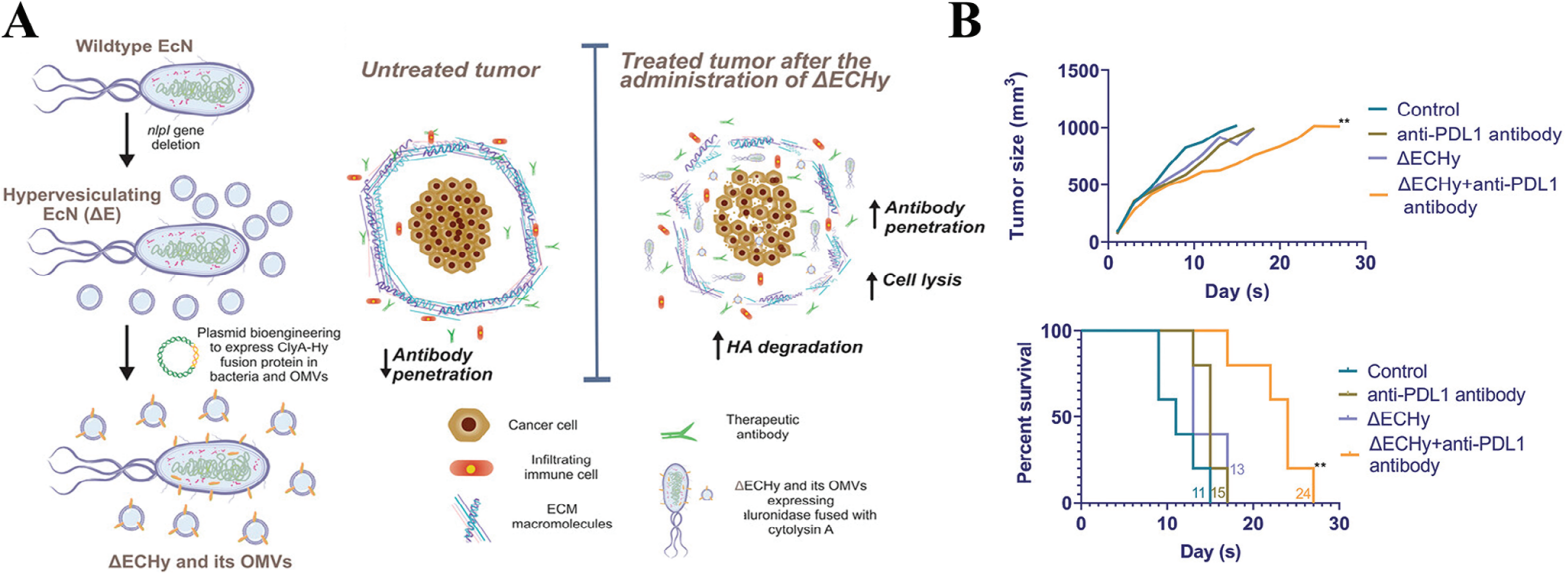
A) The production of ΔECHy and its OMVs from wild‐type EcN and mechanism of promoting tumor extracellular matrix degradation, cell lysis and antibody penetration. B) Line plot and survival rate for four groups combining ΔECHy with anti‐PDL1 antibody in MC38 tumor model. Reproduced with permission.^[^ ^99^ ^]^ Copyright 2022, Wiley‐VCH.

The tumor extracellular matrix (ECM) is a complex network of proteins, carbohydrates, and signaling molecules that surrounds and supports cells within a tumor.^[^ ^98^ ^]^ The tumor ECM can influence the immune response within the TME. Given its critical role in cancer progression, the tumor ECM has become a target for therapeutic interventions. Researchers are exploring approaches to modify the ECM to enhance drug delivery or inhibit its pro‐tumor functions. During specific research, Kotagiri et al.^[^ ^99^ ^]^ found that many solid tumors can form an extracellular matrix barrier composed of hyaluronic acid (HA) around them, making it difficult for immune cells and antibodies to enter the interior of tumor. Therefore, the author chose a probiotic *E. coli* Nissle 1917 (ΔECHy) to express a fusion of ClyA and hyaluronidase by plasmid transcription (**Figure** **4**), which degraded the tumor extracellular matrix by in situ secreted OMVs carrying hyaluronidase on its surface, thus enhancing the penetration of immune cells and activating strong immune responses. Also, thanks to the tendency of bacteria to colonize in a hypoxic and immunodeficient environment, active targeting of engineered OMVs to tumors can be achieved. To further improve tumor treatment efficacy, the authors combined ΔECHy with anti‐PD‐L1 antibodies for synergetic therapy. Experimental data revealed that compared to anti‐PDL1 antibodies group and ΔECHy group, anti‐PD‐L1 antibodies combination treatment with ΔECHy did retard tumor growth rate and showed a significant promotion in survival, suggesting that remodeling tumor stroma can effectively promote therapeutic drug immune checkpoints and subsequent immune cell infiltration to improve the efficacy of tumor immunotherapy. In addition to ECM degradation, Zhou et al.^[^ ^100^ ^]^ improved drug penetration in tumor tissues by introducing the tumor‐targeting peptide LyP1. Through gene editing, LyP1 was expressed on the surface of OMVs to enhance the internalization by tumor cells, while PD‐1 plasmids were loaded into the OMVs. The constructed LyP1‐OMV@PD‐1 induced tumor cells to express PD‐1 through the release of PD‐1 plasmids, thereby achieving self‐blockade of the PD‐1/PD‐L1 pathway.

#### 4.1.2 Immunotherapeutic Agent

**Figure 5:**
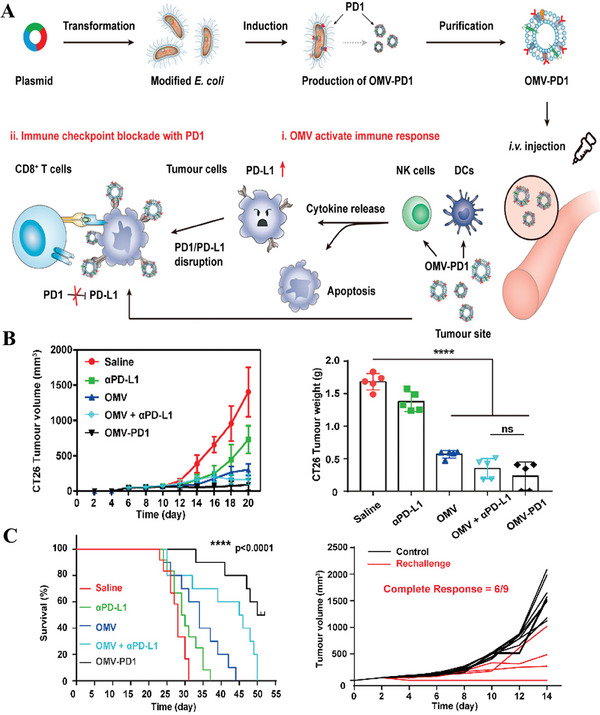
A) Graphic procedure showing gene‐modified plasmid was transformed into *E. coli* to secrete expressing PD‐1 OMVs to obtain OMV‐PD1 by purification. B) Tumor volume and final tumor weight of CT26 tumor‐bearing mice after different treatments in 20 days. C) Survival rate in 55 days of CT26 tumor‐bearing mice with different treatments; tumor volume of rechallenge with CT26 tumor cells injection to assess systematic immunity in OMV‐PD1‐cured CT26 tumor‐bearing mice at day 55. Reproduced with permission.^[^ ^103^ ^]^ Copyright 2020, American Chemical Society.

In the TME, PD‐1 and PD‐L1 are a typical pair of immune checkpoints.^[^ ^101^ ^]^ During tumor development, high expression of PD‐L1 on the surface of tumor cells binds to the PD‐1 receptor of T cells, inhibits its immune function and allows tumors to escape from immune monitoring and killing. Therefore, PD‐L1 inhibitors binding PD‐L1 on the surface of tumor cells were used to block the immune escape PD‐1/PD‐L1 pathway and restore the immune activity of T cells, thus significantly enhancing immune response^[^ ^102^ ^]^. Nie and Zhao's group^[^ ^103^ ^]^ employed this strategy to create engineered OMV‐PD1 via fusing coding region for mouse PD‐1 ectodomain with surface protein ClyA to express PD‐1 ectodomain on the surface of OMVs (**Figure** **5**). Remarkably, this ingenious genetic engineering approach maintains the OMVs' intrinsic capacity to kickstart immune activation. Of paramount significance, the engineered OMV‐PD1 exhibits an affinity for PD‐L1 on the surface of tumor cells, thus facilitating their internalization and subsequent reduction. Consequently, this process plays a pivotal role in shielding T cells from the inhibitory effects mediated by PD‐1/PD‐L1 immune axis. Through the synergistic orchestration of immune activation and checkpoint inhibition, OMV‐PD1 induced the accrual of effector T cells within the TME. This concerted action resulted in a more pronounced suppression of tumor growth in comparison to native OMVs and even the conventionally employed PD‐L1 antibody. In summary, this investigation emphasized the potential of bioengineered OMVs as highly effective immunotherapeutic agents, capable of orchestrating a comprehensive modulation of the tumor immune milieu, thereby significantly augmenting their antitumor efficacy.

This successful practice has advanced the development of bioengineered OMVs as immunotherapeutic agents. Ning et al.^[^ ^104^ ^]^ utilized genetic engineering to transcribe the genes of recombinant human tumor necrosis factor‐related apoptosis‐inducing ligand (TRAIL) into *E. coli*, resulting in the isolated‐OMVs expressing TRAIL. These OMVs were then attenuated and surface‐modified with synthesized α_v_β_3_ integrin targeting ligand (RGP) and indocyanine green (ICG) to construct I‐P‐OMVs. Experimental results revealed that I‐P‐OMVs, when activated by near‐infrared (NIR) light, could release TRAIL. This triggered apoptosis in tumor cells, effectively enhancing phototreatment and sensitizing resistant tumor cells to TRAIL therapy.

#### 4.1.3 Tumor Vaccine

In the long quest to fight cancer, researchers also began to address cancer prevention and prognostic control, such as the development of tumor vaccines. Tumor vaccine, also known as cancer vaccine or therapeutic cancer vaccine, is a type of immunotherapy designed to stimulate the body's immune system to recognize and target cancer cells.^[^ ^105^ ^]^ The fundamental principle underpinning tumor vaccines revolves around leveraging the innate capability of the immune system to detect and eradicate cancer cells. Compared with conventional treatments, tumor vaccines offer heightened dependability and efficacy due to several factors:

1. Tumor vaccines possess the capability to meticulously pinpoint suitable antigenic targets, thereby initiating fresh, precise T‐cell responses.^[^ ^106^ ^]^
2. Tumor vaccines can magnify pre‐existing tumor‐specific T‐cell responses.^[^ ^107^ ^]^
3. Tumor vaccines can expand the range and variety of tumor‐specific T‐cell responses.^[^ ^108^ ^]^
4. Within the context of tumor vaccines, the presence of specific T cell‐mediated responses and antigen memory offers distinct advantages in scenarios involving cancer recurrence.^[^ ^109^ ^]^

But even so, current cancer vaccines still suffer from some obvious disadvantages, such as low immunogenicity, complicated production, difficulty and high cost of development, and most importantly rising demand for vaccine carriers.

**Figure 6:**
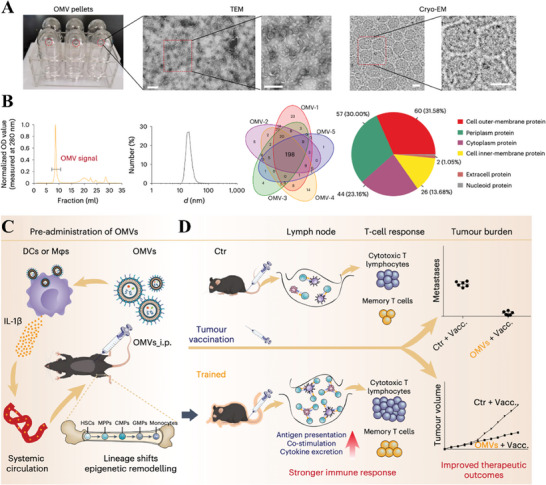
A) Transmission electron microscope (TEM) and cyrogenic electron microscope (cryo‐EM) images of isolated OMVs. B) SEC, dynamic light scattering (DLS), and proteomic analysis of OMVs. C) The process of trained immunity induced by pre‐inoculation with OMVs. D) The differences in tumor growth curves between control group mice and OMVs‐trained group mice. Reproduced with permission.^[^ ^111^ ^]^ Copyright 2023, Springer Nature.

Over the last decades, OMVs have emerged prominently as a foundational element in innovative approaches to cancer therapy. Compared with live or attenuated bacteria, OMVs have been regarded as a secure option, given their inability to self‐replicate within the host organism. Notably, OMVs display exceptional resilience to temperature variations and harbor a multitude of immunogenic elements. These elements hold the potential to incite a generalized immune response within host cells. It is these inherent attributes that position OMVs as highly suitable candidates for roles spanning from vaccines to immune modulators in pioneering cancer treatment strategies.^[^ ^110^ ^]^ According to existing studies, OMVs are suitable as an auxiliary component of tumor vaccines. On the one hand, tumor vaccination can be enhanced by pre‐inoculation with OMVs, which is a process known as trained immunity. Nie and Zhao's group has been engaged in the research of tumor vaccines based on OMVs for a long time, effectively improving the immunogenicity of tumor antigens and immunotherapy effect of tumor vaccines. In the latest study, they made exciting progress in immuno‐preactivation based on OMVs for enhancing the efficacy of tumor vaccines. They found that injection of OMVs one week before tumor vaccination significantly enhanced antitumor immunity in mice, thanks to the trained immunity resulting from pre‐inoculation with OMVs.^[^ ^111^ ^]^ This study demonstrated that OMVs induced IL‐1β secretion by activating the inflammasome signaling pathways, and the released IL‐1β entered the bone marrow and altered innate immune function, thus exhibiting enhanced immune responses to subsequent tumor vaccination (**Figure** **6**).

On the other hand, OMVs themselves have the potential to be developed into vaccines. To be gratified, the OMVs‐based vaccine Bexsero has demonstrated its feasibility in practical applications and has been approved by the FDA for clinical prevention of meningitis B in places like Brazil^[^ ^112^ ^]^ and New Zealand.^[^ ^113^ ^]^ It's not the only case that the *Bordetella pertussis* (*B. pertussis*) OMVs vaccine showed coincident efficacy assessment in mice compared to the currently approved whole‐cell *B. pertussis* vaccine.^[^ ^114^ ^]^ In conclusion, OMVs are promising targets for vaccination because of their immunogenic nature, stability in various chemical and physical conditions, low cellular toxicity, and diverse synergetic therapy strategy.^[^ ^115^ ^]^ Antitumor vaccines that harness OMVs primarily rely on advanced genetic engineering techniques. These methods entail the introduction of antigens into the vesicle lumen or onto its membrane surface, stimulating the desired immune response without compromising the original immunogenicity or provoking adverse reactions. Notably, genetic engineering permits the incorporation of homologous or heterologous antigens sourced from various pathogens or alternative origins into OMVs. A well‐established approach involves the fusion of an antigen with a protein native to OMVs, exemplified by ClyA and hemoglobin protein (Hbp). This fusion results in a chimeric protein that finds expression on the membrane of OMVs.^[^ ^116^ ^]^

**Figure 7:**
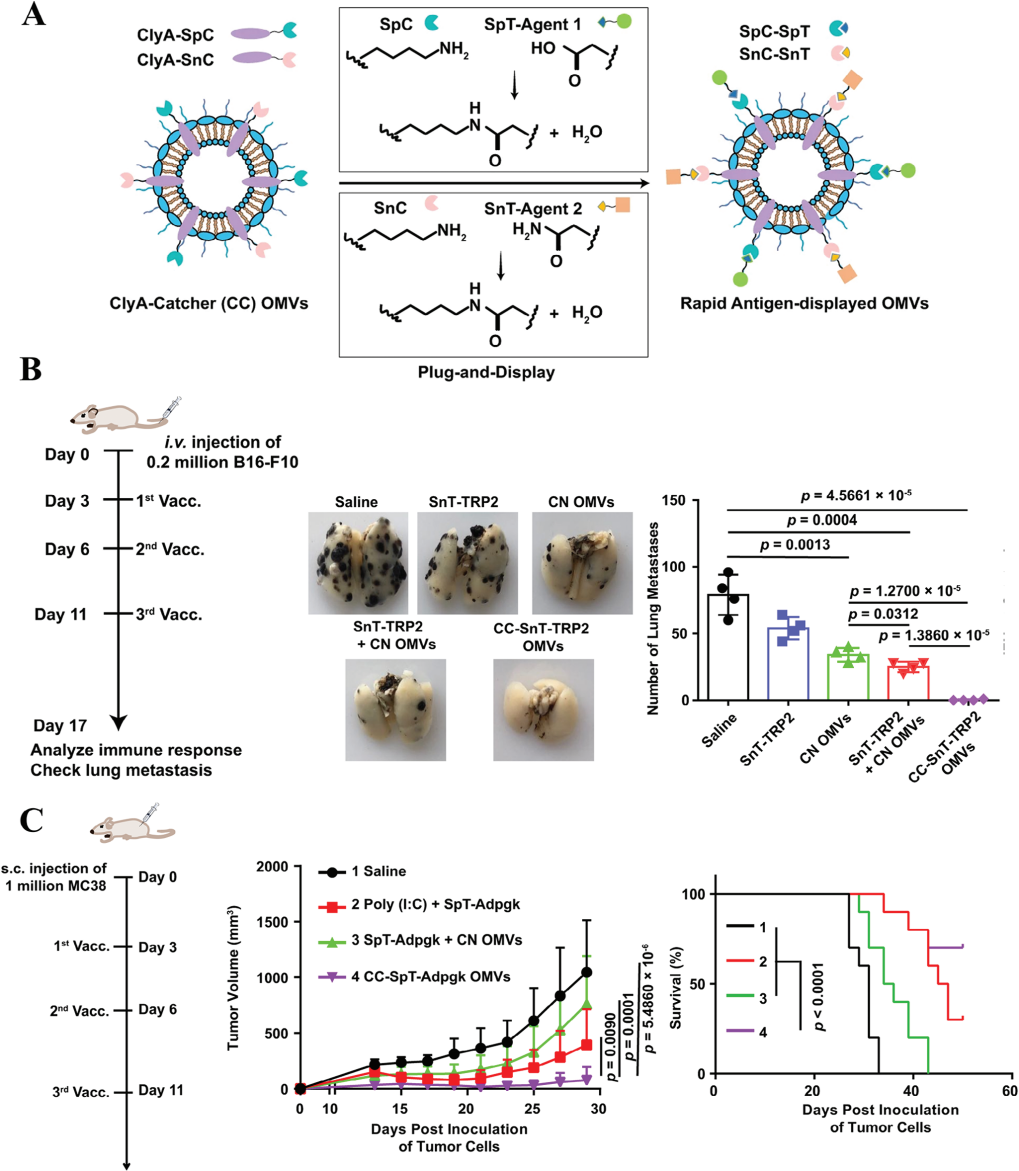
A) The mechanism of principle of ClyA catcher (CC) OMVs to display antigen rapidly by forming amido bonds. B) Experimental scheme and results of vaccination of TRP2_180‐188_ antigen‐displayed OMVs to suppress B16F10 melanoma lung metastasis. C) Experimental scheme and results of vaccination of SpT‐labeled Adpgk antigen‐displayed OMVs to inhibit MC38 tumor growth and increase survival rate. Reproduced with permission.^[^ ^117^ ^]^ Copyright 2021, Springer Nature.

In a prior investigation, Nie and Zhao's group discovered that antigens readily form a fusion with the ClyA protein located on the surface of OMVs. However, due to tumor heterogeneity, variations in genetic and phenotypic profiles exist among different tumor cells, resulting in diverse tumor antigens that can differ significantly between patients. This variability makes it impractical to develop a single OMV‐based tumor vaccine decorated with a specific antigen that would be universally effective for all patients. Therefore, it is imperative to design a versatile OMVs platform capable of rapidly and simultaneously presenting multiple antigens for the personalized development of tumor vaccines. Fortunately, their research group endeavored to create a flexible tumor vaccine platform known as CC‐OMVs (**Figure** **7**), which employs genetic engineering and “Plug‐and‐Display” technology involving tag/catcher protein pairs to display target antigens.^[^ ^117^ ^]^ Taking into account the size restrictions imposed by the Hbp surface display protein and the steric hindrance caused by the N‐ and C‐terminals of outer membrane protein A (OmpA), the authors opted for the C‐terminals of ClyA as an anchor site for the display of exogenous antigens. Experimental results show that the OMVs‐based tumor vaccine platform reliably incorporates antigens and efficiently accumulates them in lymph nodes. This suggests that OMVs‐based tumor vaccines can proficiently deliver antigens to lymph nodes, facilitating their presentation to DCs and subsequently eliciting anti‐cancer immune responses mediated by antigen‐specific T lymphocytes. This efficacy was further validated in anti‐tumor experiments conducted in mice bearing tumors.

### 4.2 Surface Functionalization of OMVs for Stimuli‐Responsive Cancer Therapy

Although applying bioengineering methods for OMVs modification appears to be a good strategy, it suffers from high cost, complex design, and a long development period. In this regard, scientists have turned their attention to surface functionalization with higher efficiency. Two main methods have been developed, namely chemical bond coupling and electrostatic adsorption.

1. Chemical bond formation involves the attachment of specific molecules or ligands to the OMVs surface through covalent bonds. Common functional groups include amino groups (‐NH_2_), carboxyl groups (‐COOH), azide groups (‐N_3_), and thiol groups (‐SH). In addition, the utilization of cross‐linking reagents may facilitate the formation of covalent bonds. These reagents have functional groups that react with the functional groups on OMVs surface. For example, amine‐reactive cross‐linkers like N‐hydroxy succinimide (NHS) esters can form stable amide bonds with amine groups on the surface of OMVs.
2. Electrostatic adsorption relies on the electrostatic attraction between positively charged molecules and negatively charged bacterial cell walls. It is based on non‐covalent interactions, usually without the involvement of chemical bonds. The surface of OMVs often has a net negative charge due to the presence of negatively charged molecules such as LPS. This strategy is commonly used for attaching positively charged molecules, including peptides, polymers, or proteins to the surface of OMVs.

**Figure 8:**
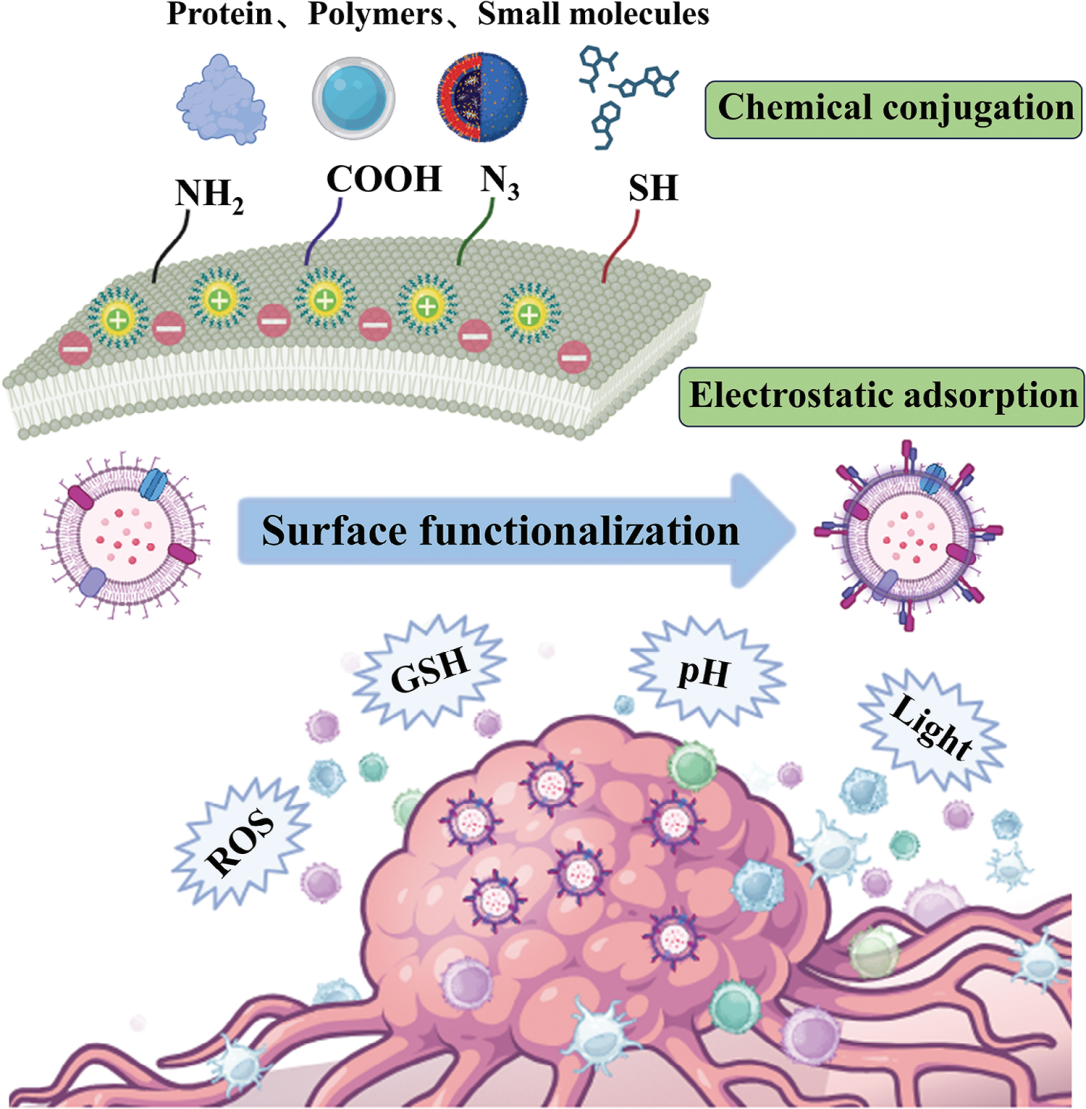
Schematic illustration of two approaches to OMVs surface functionalization for stimuli‐responsive cancer therapy. Proteins, polymers, and small molecules bind to functional groups such as ─NH_2_, ─COOH, ─N_3_, and ─SH through chemical conjugation, while electrostatic adsorption relies on the electrostatic attraction between positively charged molecules and negatively charged bacterial cell walls. Then surface‐functionalized OMVs respond to ROS, GSH, pH, and light in TME, which is expected to promote cancer therapy.

It's well known that TME has many characteristics that are different from normal tissues, such as hypoxia,^[^ ^118^ ^]^ acidity,^[^ ^119^ ^]^ reactive oxygen species (ROS),^[^ ^120^ ^]^ glutathione (GSH),^[^ ^121^ ^]^ and enzymes,^[^ ^122^ ^]^ etc. Considering the complexity of TME, stimuli‐responsive strategies^[^ ^123^, ^124^ ^]^ have been used for surface‐functionalized OMVs to achieve specific tumor therapy and reduce toxic side effects on healthy tissues (**Figure** **8**). Hence, stimuli‐responsive OMVs by surface functionalization that respond to internal or external stimuli such as ROS, GSH, pH, and light, are expected to be a powerful tool for improving cancer treatment.

**Figure 9:**
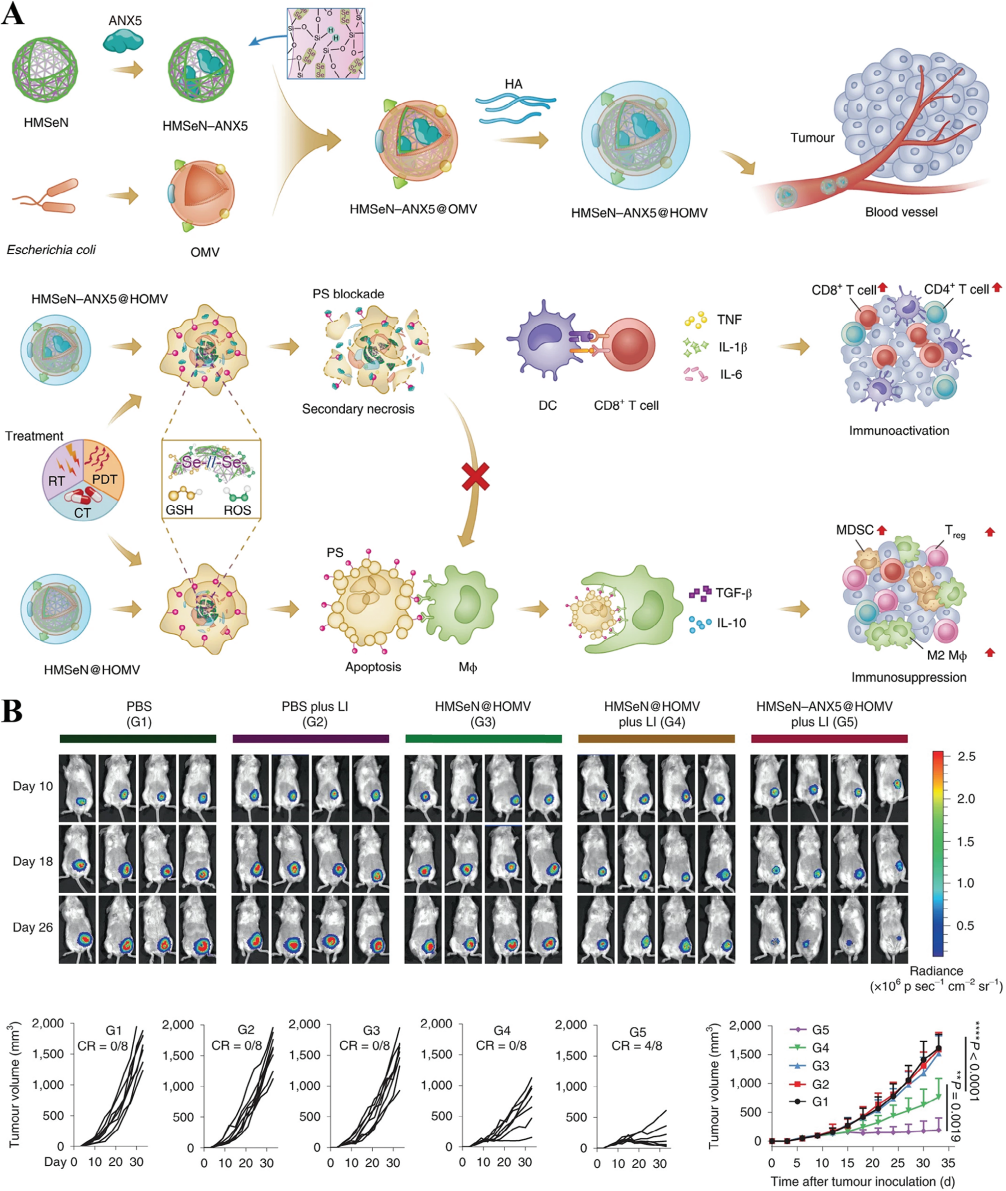
A) Schematic illustration of the preparation of HMSeN–ANX5@HOMV by coating ANX5‐immobilized diselenide‐bridged HMSeN and modification with hyaluronate on the surface of OMV. After treatment, HMSeN–ANX5@HOMV disintegrated in TME and the released ANX5 protein served as an immunomodulator through competitive blockage with PS, thus provoking anti‐tumor immune responses. B) In vivo bioluminescence imaging and tumor growth of 4T1‐Luc tumor‐bearing mice. Reproduced with permission.^[^ ^125^ ^]^ Copyright 2020, Springer Nature.

Among these stimuli, both GSH and ROS upgrade in TME. Inspired by this, Chen and Yang's group^[^ ^125^ ^]^ has made excellent work through ingenious design. They constructed a diselenide‐bridged HMSeN‐ANX5@HOMV multifunctional nanoplatform, which degraded and evoked on‐demand burst release of annexin A5 (ANX5) in both oxidative and reductive conditions of TME. As a molecular imaging agent, ANX5 is widely used as phosphatidylserine binding to detect apoptosis. As shown in **Figure** **9**, blocking the exposure of phosphatidylserine (PS) can inhibit macrophage phagocytosis and result in secondary necrosis and the in situ release of tumor‐associated antigens, thereby inducing tumor‐specific immune activation. Meanwhile, they ingeniously incubated hyaluronate with OMVs to promote the stability of HMSeN‐ANX5@HOMV in systemic circulation because of the good biocompatibility and biodegradability of HA.

**Figure 10:**
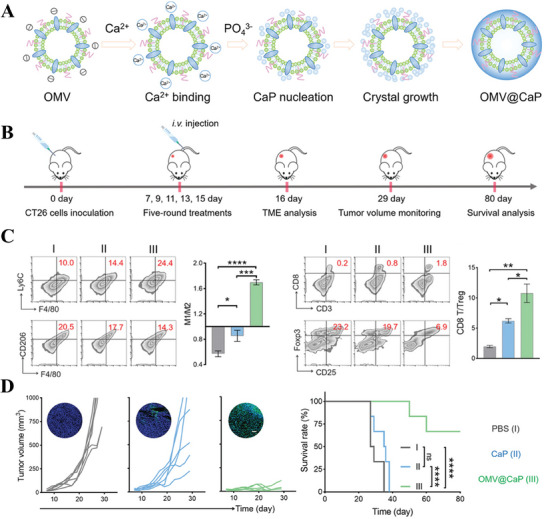
A) The preparation procedure of biomineralized OMV@CaP by electrostatic adsorption. B) Experimental protocol to assess the TME regulation and tumor suppression. C) Quantification of M1 macrophages (upper) and M2 macrophages (lower) by flow cytometry and their ratio after five‐round treatment; Quantification of CD8^+^ T (upper) and T‐regulate cells (lower) by flow cytometry and their ratio after five‐round treatment. D) Schematic illustration of tumor volume (including TUNEL staining images) and survival rate in CT26 tumor‐bearing mice. Reproduced with permission.^[^ ^126^ ^]^ Copyright 2020, Wiley‐VCH.

One of the significant features of TME is its weak acidity. This acidic microenvironment not only promotes tumor progression but also plays a role in immune escape, drug resistance, and other hallmarks of cancer. Consequently, understanding and targeting the acidic TME have become important strategies in cancer therapy research. Therefore, Ma et al.^[^ ^126^ ^]^ adopted highly biocompatible calcium phosphate (CaP) for biomineralization modification of OMVs as shown in **Figure** **10**. The resulting nanoparticles OMV@CaP not only avoid excessive inflammatory response induced by wild‐type OMVs, reducing severe side effects, but also significantly improve tumor suppression with the help of the pH‐sensitive CaP group to neutralize the slightly acidic tumor site, leading to the exposure of tumor to immune cells. In terms of in vitro experiments, OMV@CaP can not only significantly promote the polarization of M2‐to‐M1 macrophages and even reverse the ratio of two phenotypes, but also increase the secretion of CD8^+^ T cells and inhibit the secretion of immunosuppressive Treg cells, suggesting effective activation of immune responses. Meanwhile, OMV@CaP can almost completely inhibit tumor growth and prolong the life‐span of tumor‐bearing mice in vivo. The innovation of this study is to inspire us that apart from chemical bond coupling, the negative electronegativity of bacterial membrane can also be used as an entry point to achieve efficient functionalization by classical electrostatic adsorption, and biomineralization is a highlight of that.

**Figure 11:**
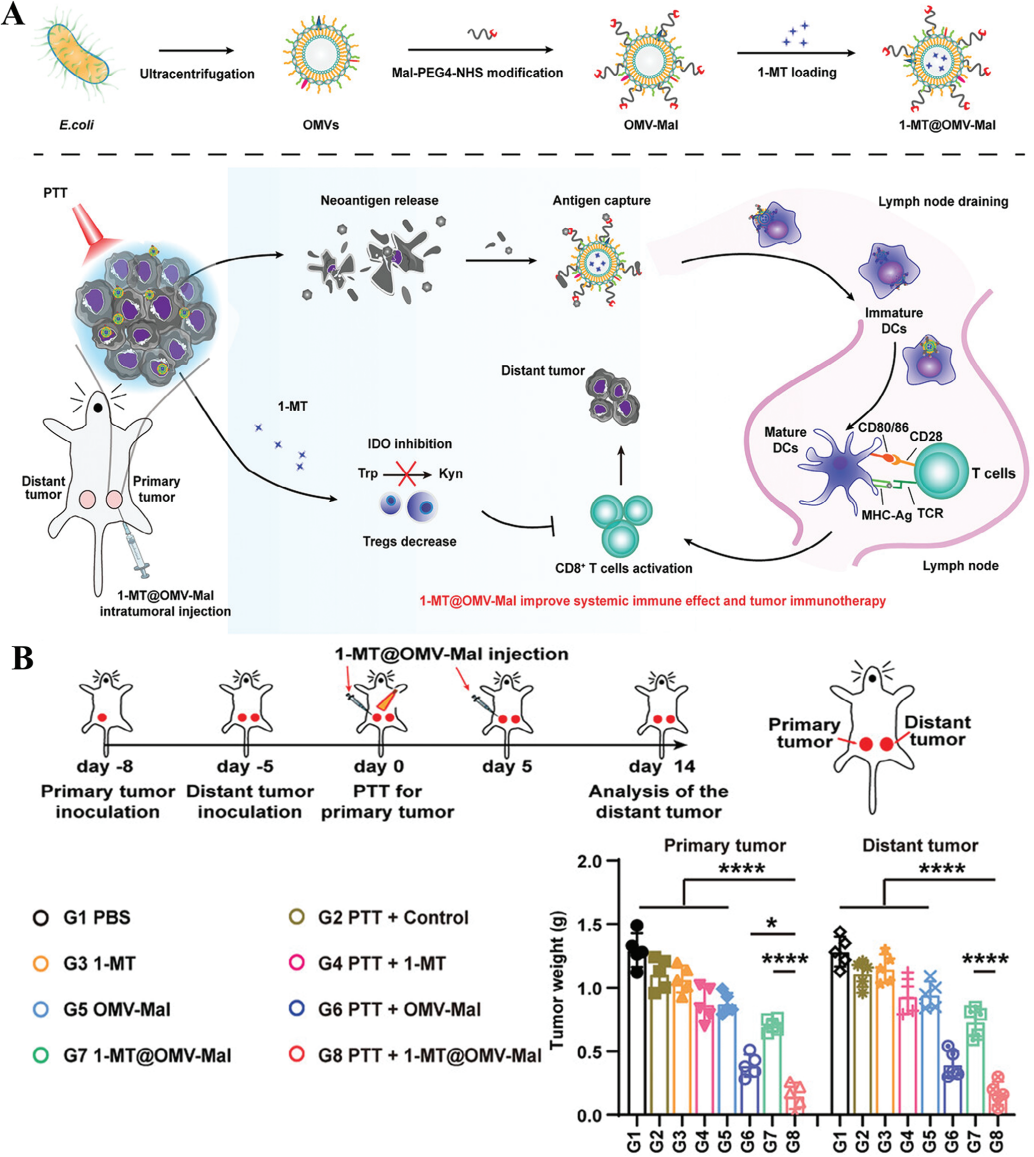
A) The preparation of 1‐MT@OMV‐Mal including ultracentrifugation, Mal‐PEG4‐NHS modification, and 1‐MT loading and graphic abstract of 1‐MT@OMV‐Mal eliciting systemic antitumor immune responses after PTT. B) The experiment design of primary tumor and distant tumor model and evaluate both of their tumor weight changes after 1‐MT@OMV‐Mal injection at day 14. Reproduced with permission.^[^ ^127^ ^]^ Copyright 2022, Wiley‐VCH.

As a non‐invasive means, the external light source stimulation response has great potential for cancer treatment. Hence, Zhao et al.^[^ ^127^ ^]^ designed an OMVs‐based in situ vaccine 1‐MT@OMV‐Mal by surface chemical modification of the Mal‐PEG4‐NHS moiety as shown in **Figure** **11**. To promote the capture of tumor neoantigens, author utilized amino group on the surface of OMVs to conjugate the Mal‐PEG4‐NHS moiety, subsequently forming a stable thioether bond between the terminal NHS and antigens. Meanwhile, the inhibitor of indoleamine 2,3‐dioxygenase (IDO), 1‐methyl‐tryptophan (1‐MT) was loaded into OMVs to modulate the Tregs‐mediated TME, thereby effectively activating the immune responses. To synergistically enhance tumor treatment efficacy, Zhao et al. innovatively introduced photothermal therapy (PTT) by utilizing ICG, the clinically approved photothermal agent (PTA). Under the irradiation of NIR light, the PTA was excited to generate heat, which caused the tumor to rupture. Research data showed that neoantigens released from tumor fragments after PTT can induce specific immune responses to further clear residual lesions and prevent tumor metastasis (Figure 11B).

### 4.3 Hybrid Membrane Vesicles

Addressing the challenge of tumor heterogeneity is an unavoidable issue in antitumor research. For personalized tumor therapy, tumor cell membranes provide a promising pathway because of their ability to target tumors homologously and their abundance of tumor antigens.^[^ ^128^ ^]^ The antigens from primary tumors are evidenced to induce innate and adaptive tumor‐specific immune responses. More importantly, it can act as a good candidate for tumor vaccine development without genetic information.^[^ ^129^ ^]^ Due to these potential advantages, the innovative utilization of cancer cell‐derived membranes for various therapeutic purposes has gained attention in recent years. These membranes can be harvested from patients’ own cancer cells or cancer cell lines, and then applied to address specific challenges in cancer therapy, such as personalized treatment, drug resistance, and targeted drug delivery. A hybrid membrane system refers to a structure that combines two or more different types of membranes to create an integrated system with unique properties and enhanced functionality.^[^ ^130^ ^]^ Recently, fusing tumor cell membranes with OMVs to generate a hybrid membrane for tumor immunotherapy has become a hotspot.

**Figure 12:**
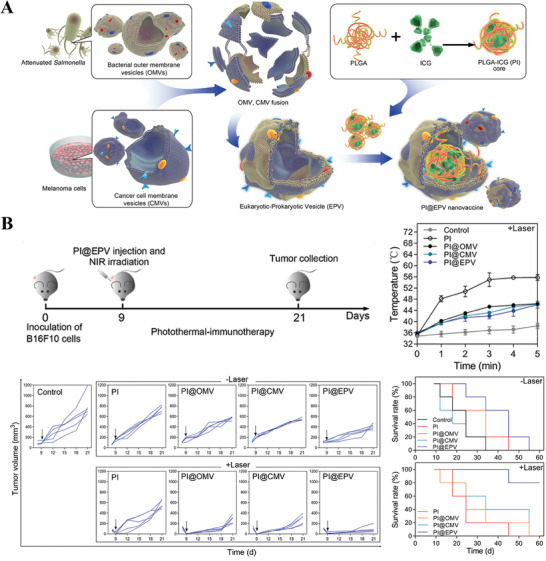
A) Schematic illustration of constituent parts and preparation procedure of PI@EPV B) Therapeutic assessment of PI@EPV in B16F10 tumor‐bearing mice involving photothermal effects and tumor growth suppression. Reproduced with permission.^[^ ^131^ ^]^ Copyright 2020, Wiley‐VCH.

Given the potential immunogenicity and homogeneous‐targeting ability of tumor cell membranes, Tang et al.^[^ ^131^ ^]^ first proposed a eukaryotic‐prokaryotic vesicle (EPV) hybridization strategy by fusing melanoma cytomembrane vesicles (CMVs) with attenuated *Salmonella* OMVs in 2020 (**Figure** **12**). This design allowed EPV to integrate melanoma antigens with natural adjuvants to form a single therapeutic agent. Meanwhile, the authors introduced ICG, the FDA‐approved photosensitizers (PTS), to induce PTT, resulting in synergistic antitumor effects in the melanoma model. As illustrated in Figure 12, B16F10 tumor‐bearing mice were irradiated by NIR light and their rising body temperature revealed the occurrence of photothermal effects. With injection of PI@EPV at day 9, on one hand, PI@EPV without laser group showed strong tumor inhibition in comparison to control group but failed to completely suppress tumor growth; on the other hand, compared to PI@EPV without laser group, PI@EPV with laser group exerted more significant efficacy in tumor treatment, fully demonstrating the power of a combination of PTT and immunotherapy based on hybrid membrane systems.

**Figure 13:**
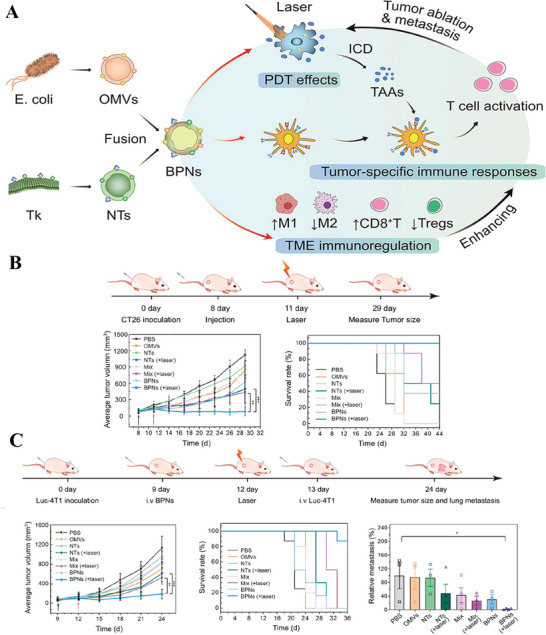
A) OMVs and NTs were fused to generate hybrid membrane vesicles BPNs, showing their functions in photodynamic therapy, tumor‐specific immune responses and TME immunoregulation. B) The experiment process of CT26 tumor‐bearing mice therapy model based on average tumor volume and survival curve (black and red arrows indicate time nodes of intravenous administration and laser irradiation, respectively). C) The experiment process of Luc‐4T1 tumor‐bearing mice lung metastasis model and assessment indicators within three dimensions including average tumor volume, survival rate and quantitative analysis of relative metastasis (black and red arrows indicate time nodes of intravenous administration and laser irradiation, respectively). Reproduced with permission.^[^ ^133^ ^]^ Copyright 2022, American Chemical Society.

Similarly, Zhang et al.^[^ ^132^ ^]^ reported functional hybrid membrane mTOMVs, which were made up of OMVs and tumor‐derived cell membranes, with the function of targeting tumor and immune activation. Notably, this nanoplatform evoked strong adaptive immune responses against homologous tumors rather than heterogeneous tumors. Although current OMVs‐based membrane systems integrating with tumor cell membranes are effective in stimulating antigen‐specific immune responses, this strategy still has some limitations. For example, different tumors have different antigenic phenotypes, so a single‐mode of tumor antigen is not universal to different cancer treatments. Therefore, newly emerging thylakoid membranes extracted from plants is a novel avenue for enhanced tumor treatment. Xie et al.^[^ ^133^ ^]^ creatively developed a strategy that involves the fusion of OMVs with thylakoid nanovesicles (NTs) to create hybrid vesicles known as bacteria‐plant hybrid vesicles (BPNs). These BPNs exhibit phytochemical properties, leading to enhanced photodynamic effects for immunotherapy (**Figure** **13**). The researchers investigated the molecular mechanism underlying the accumulation of administered BPNs in tumor tissue and their subsequent role in inducing immune responses. This includes the activation of immune cells and the production of cytokines. Moreover, with the features of abundant photosystem‐associated enzymes, NTs can produce singlet oxygen (^1^O_2_) upon laser irradiation to kill local tumors, release tumor‐associated antigens, and further significantly boost immune activation. In vivo experimental data revealed that compared to other groups, whether in tumor suppression or metastatic tumor models (Figure 13B,C), BPNs with laser group demonstrated the strongest tumor suppression, in which the photodynamic effect played a crucial role. Overall, hybrid membrane vesicles‐based BPNs were capable of inhibiting tumor growth and preventing metastasis without side effects.

### 4.4 Drug Delivery Capacity of OMVs for Combination Therapy

In addition to hybrid membrane construction, there has been a hot area over the past decade that OMVs are increasingly attractive to be outstanding drug delivery vectors.^[^ ^134^ ^]^ Attributed to lipid phospholipid membranes, OMVs can fuse with the host cell membrane or enter directly, whereas entry into immune cells occurs through endocytic and phagocytic pathways.^[^ ^135^ ^]^ Besides, OMVs allow the incorporation of hydrophobic drugs into the lipophilic leaflets,^[^ ^136^ ^]^ which enables the transportation of diverse bioactive molecules to recipient cells, thereby influencing their physiological activity. Thanks to their capacity to encapsulate and transport an array of bioactive compounds, nanoscale OMVs hold significant promise as an emerging class of drug delivery vehicles. Furthermore, researches have confirmed that OMVs possess the capability to infiltrate remote organs via systemic circulation. In 2020, Ning et al.^[^ ^104^ ^]^ reported a novel OMVs‐based drug delivery system I‐P‐OMVs, which exhibited excellent stratum corneum penetration and specifically targeted to melanoma through the lipid fusion effect. This occurs particularly under conditions of microbial imbalance and within an inflammatory milieu, where tight junctions become compromised.^[^ ^137^ ^]^ Given the advantages of drug delivery, various OMVs‐based drug delivery platforms have been explored for cancer immunotherapy. For example, Ping et al.^[^ ^138^ ^]^ proposed a biomedical approach where OMVs‐derived from attenuated *Salmonella* were co‐extruded with DSPE‐PEG‐RGD (OR) to generate a tumor‐targeting polymeric micelle. In order to achieve synergistic therapeutic effects, 5‐fluorouracil (5‐FU) prodrug tegafur‐loaded vector FT was coated with OR to form a “core‐shell” structure of nanomedicine ORFT. Moreover, OMVs excel as vehicles for targeted delivery, facilitated by their interaction with neutrophils. Obstructed by the blood brain barrier (BBB), many brain‐in‐use drugs cannot work well because of the restriction on entering the brain.^[^ ^139^ ^]^ Recent studies have found that neutrophils can mediate enhanced drug accumulation in the brain. To overcome the efficiency of targeting delivery, Rong et al.^[^ ^81^ ^]^ reported a *Salmonella*‐based drug delivery system OMVs/DOX for enhancing the effectiveness of glioma chemotherapy. They adopted the anaerobic bacteria *Salmonella* as a “Trojan horse”, OMVs/DOX showed remarkable tumor homing ability by virtue of selective colonization and reproduction in hypoxic cancer cells. Meanwhile, the PAMPs on the surface of OMVs/DOX can be recognized by TLR4 in neutrophils and promote subsequent endocytosis by neutrophils. With the assistance of neutrophil‐mediated hitchhiking drug delivery, OMVs/DOX are capable of crossing the BBB and localizing to tumor tissue, achieving in situ release of the antitumor drug Doxorubicin (DOX).

However, due to the immunosuppressive effect of TME, single immunotherapy has an unsatisfactory therapeutic outcome. Consequently, the utilization of OMVs‐based delivery systems has sparked a creative collaboration in interdisciplinary research aimed at improving cancer immunotherapy.^[^ ^140^, ^141^ ^]^ This includes synergistic approaches involving chemodynamic therapy (CDT), photodynamic therapy (PDT), and PTT. With the influence of combination therapy, immunogenic cell death (ICD) and other cell apoptosis pathways can be activated to promote the infiltration of immune cells around tumors, thus reprogramming TME and eliciting immune responses.

#### 4.4.1 Combination with PDT/PTT

**Figure 14:**
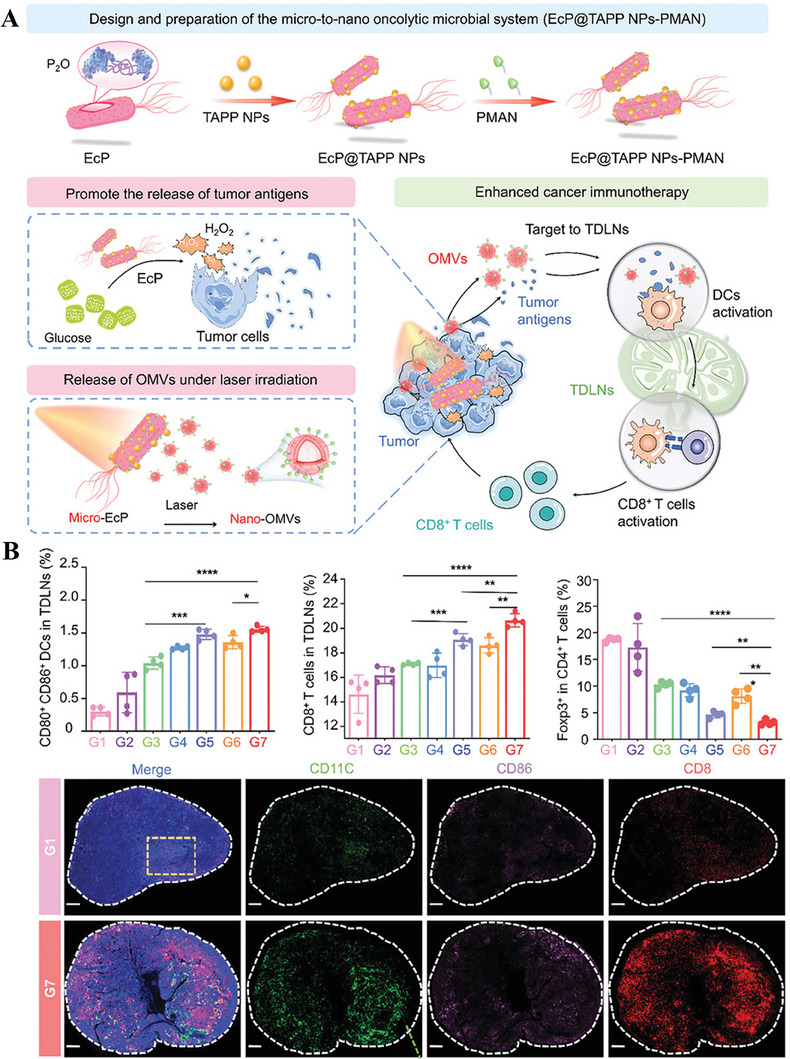
A) Graphic illustration of the preparation and therapeutic strategy of EcP@TAPP NPs‐PMAN by promoting tumor antigens release and OMVs secretion under light irradiation. B)The proportion of CD80^+^ CD86^+^ DCs, CD3^+^ CD8^+^ T cells, Tregs, and CLSM image in the TDLNs tissue after different treatments. DAPI (blue), anti‐CD11c antibody (green), anti‐CD86 antibody (purple), and anti‐CD8 antibody (red). G1: PBS, G2: TAPP NPs+laser, G3: EcP, G4: EcP@TAPP NPs, G5: EcP@TAPP NPs+laser, G6: EcP@TAPP NPs‐PMAN and G7: EcP@TAPP NPs‐PMAN+laser. Reproduced with permission.^[^ ^145^ ^]^ Copyright 2023, Wiley‐VCH.

PDT is a process of using light sources to stimulate PTS to produce non‐radiative transitions, thus converting surrounding oxygen into ROS to kill tumor cells.^[^ ^142^, ^143^ ^]^ In preliminary evaluations, Xiang et al.^[^ ^144^ ^]^ found that OMVs showed potent antitumor potentials, but the therapeutic window is narrow. To enhance the antitumor efficacy, they encapsulated photosensitizer chlorin e6 (Ce6) into OMVs to induce photodynamic‐enhanced immunotherapy. Research data showed that Ce6@OMVs through macrophages uptake promoted M2‐to‐M1 macrophages repolarization and ROS was produced under external light irradiation to induce ICD, which remarkably demonstrated OMVs are promising as platforms in combination with other strategies for synergic antitumor therapy. By constructing a laser irradiation‐triggered OMVs release system as shown in **Figure** **14**, Sun et al.^[^ ^145^ ^]^ further verified this conclusion. In ideal conditions, the released antigens from tumors can be recognized by DCs and presented to tumor‐draining lymph nodes (TDLNs), evoking tumor‐specific immune responses. However, TDLNs are usually in an inactivation state owing to TME. To handle this challenge, Sun et al. incubated porphyrin derivatives (TAPP) and PEG‐capped 4‐aminophenyl‐d‐mannopyranoside (PMAN) with pyranose oxidase (P_2_O) expressing engineered *E. coli*, which not only promote P_2_O‐catalyzed ROS production to release tumor‐associated antigens, but also induce OMVs secretion under light irradiation. With reciprocal effect, OMVs and tumor antigens effectively activate the antigen presenting ability of DCs, thus restoring the immunoactive state of TDLNs.

**Figure 15:**
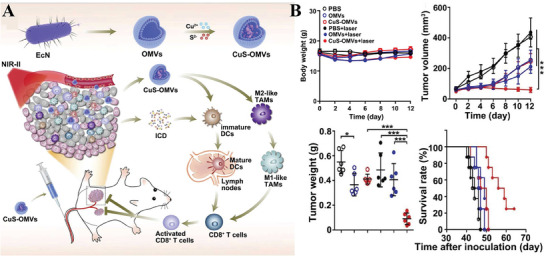
A) Graphic presentation of the fabrication of CuS‐OMVs and its synergistic photothermal‐induced immunotherapy mechanisms of immune responses activation by DCs maturation and M2‐to‐M1 macrophages repolarization. B) In vivo antitumor efficacy of different treatment groups. Reproduced with permission.^[^ ^150^ ^]^ Copyright 2022, Elsevier.

Though, PDT still has some limitations such as oxygen dependence.^[^ ^146^ ^]^ Increasing attention has been paid to the researches of PTT, which harnesses the PTA to convert light energy into heat energy, so that the temperature of the tumor site can be selectively increased, achieving the purpose of tumor ablation.^[^ ^147^, ^148^ ^]^ Therefore, PTT is widely used in the treatment of skin melanoma owing to its advantages of being time‐ and space‐controlled, low side effects and short‐time treatment. For example, Zhang et al.^[^ ^149^ ^]^ selected hollow polydopamine (HPDA) nanoparticles as PTA and ingeniously encapsulated them into OMVs to induce PTT for melanoma treatment. However, because of the limited penetration of external light and the poor targeting of PTA, the therapeutic outcome of melanoma located in deep skin tissue is unsatisfactory. Inspired by this, Gan et al.^[^ ^150^ ^]^ adopted copper sulfide (CuS) nanoparticles as a powerful PTA, whose absorbance is in the NIR‐II region that allows deeper tissue penetration and less toxic side‐effects. For synergistic photothermal‐immunotherapy, they developed prospective biomimetic nanomaterials CuS‐OMVs as shown in **Figure** **15**. On the one hand, they smartly utilized OMVs as drug delivery carriers for enhanced tumor targeting capability; on the other hand, they efficiently fabricated CuS with an intelligent use of OMVs as the nanoreactor template by one‐spot method. Results revealed that CuS‐OMVs accumulated at the tumor site after intravenously injection and in situ generated hyperthermia to ablate tumors under NIR‐II light irradiation. The release of tumor fragments further caused ICD effects to promote anticancer efficacy through DCs maturation and M2‐to‐M1 macrophages repolarization.

#### 4.4.2 Combination with CDT

**Figure 16:**
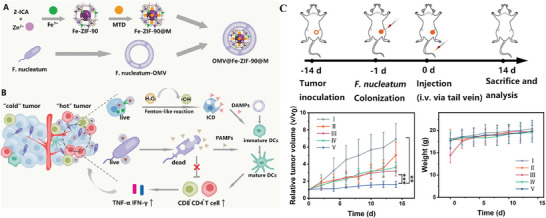
A) The preparation of OMV@Fe‐ZIF‐90@M by co‐loading Fe‐ZIF‐90@M with *F. nucleatum*‐derived OMVs. B) The mechanism of activated two cell pathways promotes DCs maturation, thus transforming “cold” tumors into “hot” tumors. C) In vivo antitumor experimental scheme and results from different treatment groups. I: PBS, II: MTD, III: Fe‐ZIF‐90, IV: Fe‐ZIF‐90@M, V: OMV@Fe‐ZIF‐90@M. Reproduced with permission.^[^ ^153^ ^]^ Copyright 2023, American Chemical Society.

CDT is a novel tumor therapy technique that utilizes the TME to activate Fenton or Fenton‐like reactions to generate strong oxidizing hydroxyl radical (∙OH) for tumor‐specific treatment.^[^ ^151^ ^]^ Compared with PDT and PTT, CDT does not rely on oxygen inside the tumor and external light sources, and is suitable for deep tissue therapy, showing its good application prospects in tumor therapy.^[^ ^152^ ^]^ Qu et al.^[^ ^153^ ^]^ proposed an OMVs‐coated nanoplatform OMV@Fe‐ZIF‐90@M that combines immunotherapy with chemodynamic therapy as shown in **Figure** **16**. Based on the finding that *Fusobacterium nucleatum* (*F. nucleatum*) colonizes triple‐negative breast tumors and promotes tumor progression and metastasis, they chose it as a target to improve the treatment of triple‐negative breast cancer (TNBC). To introduce chemodynmaic effects, a Fe(III)‐based metal organic framework (MOF) containing the antibiotic metronidazole (MTD) was loaded in *F. nucleatum*‐secreted OMVs. Research results showed that OMV@Fe‐ZIF‐90@M could precisely locate tumor cells through homing effect. The in situ release of MTD and Fe individually induced ICD through Fenton‐like reactions and antibacterial effects to produce damage associated molecular patterns (DAMPs) and PAMPs, thereby transforming cold tumors into hot tumors and effectively activating immune responses. Fenton or Fenton‐like reactions typically rely on the induction of iron elements.^[^ ^154^ ^]^ However, conventional MOFs often suffer from low iron content. To enhance the effective concentration of iron ions, Xie et al.^[^ ^155^ ^]^ introduced FDA‐approved natural polyphenol tannic acid (TA), which coordinated with ferric ions to form supramolecular metal‐phenolic networks (MPNs) that coated the surface of OMVs. This MPNs not only effectively released ferric ions in TME with low pH and high ATP, inducing Fenton or Fenton‐like reactions, but also facilitated dynamic changes in iron ions under the influence of TA, significantly promoting the process of CDT‐induced ICD.

**Figure 17:**
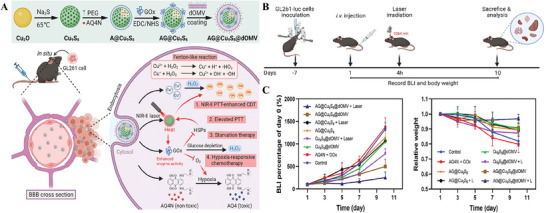
A) Schematic illustration of preparation procedure of biomimetic nanoreactor AG@Cu_9_S_8_@dOMV and its mechanisms of synergetic therapy including NIR‐II PTT‐enhanced CDT, elevated PTT, starvation therapy and hypoxia‐responsive chemotherapy. B) Schema of in vivo antitumor efficacy and toxicity evaluation in GL‐261‐luc tumor bearing mice model. C) Quantitative analysis of the bioluminescence percentage and relative body weight of treated mice on day 1, day 3, day 5, day 7, and day 10 respectively. Reproduced with permission.^[^ ^156^ ^]^ Copyright 2023, Elsevier.

The low immunogenicity of TME can be effectively improved by CDT. However, at present, CDT has the problem of low therapeutic efficiency. Thus, harnessing inorganic catalysts to generate heat under light not only induces photothermal effects, but also promotes the Fenton‐like reaction to enhance CDT. Mou et al.^[^ ^156^ ^]^ constructed LPS‐free dOMVs camouflaged nanoreactors (**Figure** **17**). First, glucose oxidase (GOx) within the nanoreactors rapidly depletes intratumoral glucose to generate hydrogen peroxide (H_2_O_2_) and gluconic acid, enabling starvation therapy. Second, abundant H_2_O_2_ induces CDT by Cu_9_S_8_‐mediated Fenton‐like reactions. Finally, the incorporation of Cu_9_S_8_ nanoparticles, with a strong NIR‐II absorption capacity, raised local tumor temperatures upon NIR‐II laser irradiation. This induced photothermal‐enhanced CDT, enhanced GOx activity, and expedited drug release.

## 5 Preclinical and Clinical Studies

Through extensive in vitro and in vivo studies, the PAMPs of OMVs have been demonstrated to have natural immunogenicity, enabling them to elicit immune responses. In the next decade, significant progress was made in the development of OMVs‐based vaccines. The first OMVs‐based vaccine Bexsero against *Neisseria meningitidis* serogroup B, was licensed and marketed in several countries.^[^ ^157^ ^]^ This marked a major milestone in translating the OMVs research into clinical use. The success of Group B meningococcal‐associated OMVs vaccines also fully confirms the safety and treatment efficiency of OMVs‐based treatment. Therefore, OMVs‐based vaccines against *Vibrio choleare* (*V. cholerae*), *S. typhimurium* and *Shigella flexneri* have been studied in animal models, but have not yet progressed to clinical trials.^[^ ^158^, ^159^ ^]^ In the latest report, GlaxoSmithKline's vaccine to prevent *Neisseria gonorrhea* (*N.gonorrhea*) was approved by the FDA for a phase II clinical study^[^ ^160^ ^]^ using OMVs known as generalized modules for membrane antigens (GMMAs). In addition, the Walter Reed Army Institute also conducted clinical studies of OMVs‐based vaccines given intranasally. The results showed that patients had increased intranasal immunoglobulin A (IgA) titers, and the vaccines were well tolerated without nasal inflammation.^[^ ^161^ ^]^ Clinical studies performed by the Norwegian National Institute of Public Health have found that serum antibodies of those vaccinated with intranasally applied OMVs are similar to those of intramuscular injections, and antibody levels can continue to rise in six months.^[^ ^162^ ^]^ Meanwhile, clinical studies on the safety and efficacy of intranasal OMVs as vectors for the Avacc 10 vaccine are underway in healthy volunteers (NCT05604690).

Attracted by the great potential of OMVs in the biomedical field, researches on OMVs in the 2010s expanded into therapeutic applications beyond vaccines, such as drug delivery, antibiotic infections,^[^ ^163^ ^]^ and cancer therapy. Over the past decade or so, researchers have promoted great advancements in using OMVs as bioactive materials with the assistance of different techniques, especially in cancer immunotherapy. Therefore, in this review, we summarize the knowledge on OMVs regarding biogenesis, preparation and roles in the TME, and discuss the latest advances in biomedical applications following diverse modifications such as bioengineered recombination, surface structural transformation, construction of hybrid membranes, and drug loading, as summarized in **Table** **3**.

**Table 3:** Applications of modified OMVs in cancer therapy in recent years.

Bacteria strain	Modification type	Biomedical applications	Tumor model	References
*E. coli* BL21	Biomineralization	Immunostimulants for tumor microenvironment reprogramming	CT26	[126]
*E. coli* DH5α	Display antigen on the surface	Specific tumor antigen‐based vaccine elicits innate and adaptive immune responses	B16F10	[164]
*E. coli* BL21	Biomineralization	Chemodynamic‐enhanced immunotherapy	CT26	[155]
*E. coli*	Inorganic nanoparticles loading	Neutrophil‐mediated targeted delivery and photothermally enhanced immunotherapy	B16F10	[165]
*E. coli* DE3	Gene recombination	Versatile tumor antigen display for synergistic immune response	MC38/B16F10	[117]
*E. coli* BL21	Drug loading and surface conjugated binding	Photothermal‐enhanced immunotherapy	CT26	[127]
*E. coli* Nissle 1917	Gene recombination	Stromal remodeling enhanced immunotherapy	MC38/4T1	[99]
*F. nucleatum*	Inorganic nanoparticles loading	Chemodynamic‐enhanced immunotherapy	4T1	[153]
*E. coli* Nissle 1917	Inorganic nanoparticles loading	Photothermal‐enhanced immunotherapy	4T1	[150]
*E. coli* BL21	Gene recombination and surface conjugated binding	TME reprogramming for enhanced immunotherapy	MC38/B16F10	[166]
*E. coli* BL21	Gene recombination	mRNA antigen display for immunotherapy	MC38/B16F10	[96]
*S. typhimurium*	Photosensitizer and drug loading	Photothermal‐enhanced immunotherapy	H22/CT26/4T1	[167]
*E. coli* DH5α	Inorganic nanoparticles and prodrug loading	Combinational chemodynamic/photothermal/starvation/chemotherapy	GL261	[156]
*E. coli*	Gene recombination	Enhanced immunotherapy after surgery	B16F10	[168]
*E. coli* MG1655	Drug loading and surface conjugated binding	TME reprogramming for enhanced immunotherapy	B16F10/CT26	[169]
*E. coli* MG1655	Fusion with plant thylakoid membrane	Photodynamic‐enhanced immunotherapy	CT26/4T1	[133]
*S. typhimurium*	Antitumor drug loading	Neutrophil‐mediated blood brain barrier delivery and enhanced chemotherapy	C6	[81]
*E. coli* DH5α	Photosensitizer and antitumor drug loading	Combinational photodynamic/chemo‐/immunotherapy	4T1	[144]
*E. coli* BL21	Gene recombination, biomineralization and drug loading	Trained immunity‐related vaccine for enhanced antitumor therapy	MC38/B16F10	[170]
*S. typhimurium*	Prodrug loading and surface fusion	Tumor targeting and combinational chemo‐immunotherapy	B16F10/4T1	[138]
*K. pneumonia*	Antitumor drug loading	Combinational chemo‐immunotherapy	A549	[171]
*E. coli* W3110	Gene recombination	TME reprogramming for enhanced immunotherapy	CT26/B16F10	[103]
*E. coli* DH5α	Gene recombination	Vaccine delivery system induce antibody production against autoantigen	B16F10	[172]
*E. coli*	Gene recombination, surface conjugated binding	Tumor targeting and photo‐TRAIL–programmed treatment	B16F10	[104]
*S. typhimurium*	Photosensitizer loading and fusion with cancer membrane	Combinational photothermal‐immunotherapy	B16F10/4T1	[131]
*E. coli* Trans1‐T1	Polymeric nanoparticles and antitumor drug loading	Active targeting and combinational photothermal‐immunotherapy	EMT6	[173]
*E. coli*	Drug loading and surface fusion	TME reprogramming for enhanced immunotherapy	4T1	[125]
*E. coli* W3110	Drug loading and gene recombination	Active targeting and gene silence for enhanced immunotherapy	HCC1954	[96]
*E. coli* MG1655	Inorganic nanoparticles loading and fusion with cancer membrane	Combinational radiotherapy and immunotherapy	4T1	[174]

Overall, there are four strategies for functionalizing of OMVs including genetic engineering, surface functionalization, drug delivery platforms, and hybrid membrane vesicles. Genetic engineering allows for extensive modifications and broad applications; however, it is costly, time‐consuming, and complex. Surface functionalization enhances tumor targeting and permeability with high efficiency and simplicity, yet it may lead to heterogeneity and reduced biocompatibility. Hybrid membrane construction enables precise functionalization for personalized treatment, though it involves complicated operations and potential structural damage. Drug loading enhances stability and bioavailability, but it is limited by low drug loading efficiency and poor release rates. Although each modification method has its own advantages and disadvantages (**Table** **4**), it can help meet different therapeutic needs in clinical practice for enhanced cancer immunotherapy.

**Table 4:** The advantages and disadvantages of different modified‐OMVs.

Modification	Advantages	Disadvantages
Genetic engineering	Abundant modification, universal applications	High cost, time consuming and complicated operation
Surface functionalization	Promoted tumor‐targeting and permeability, high efficency and simple operation	Heterogeneous modification and reduced biocompatibility
Hybrid membrane	Precise functionalizations for personalized treatment	Complicated operation and potential structural damage
Drug loading	Enhanced drug stability and bioavailability	Low drug loading efficiency and poor drug release rate

## 6 Conclusion and Perspectives

Since the discovery, OMVs have attracted significant attention due to the advantageous properties including natural origin as non‐cellular components and physiological activity in stimulating immune responses. However, the expansion of biomedical applications has been hindered by the excessive toxicity and limited functions of wild‐type OMVs. As a result, scientists have been dedicated to engineering modified‐OMVs. The necessity of modifying wild‐type OMVs is underscored by several key factors:

1. Modifying OMVs allows them to carry a variety of antigens and immunomodulatory molecules, thereby enhancing their potential in vaccine development and cancer immunotherapy. For instance, genetically or chemically modified OMVs can carry specific proteins or polysaccharides, increasing their efficacy as vaccine carriers.^[^ ^116^ ^]^
2. Modified OMVs can enhance their immunostimulatory capabilities. For example, OMVs modified with tumor antigens^[^ ^117^ ^]^ can stimulate robust antitumor immune responses, promoting the activation of DCs and CTLs, thereby inhibiting tumor growth and metastasis.
3. By fusing OMVs with tumor cell membranes to form hybrid membrane vesicles such as PI@EPV^[^ ^131^ ^]^ or mTOMVs^[^ ^132^ ^]^, personalized tumor vaccine development can be achieved. These modified OMVs not only provide immune stimulation but also carry tumor‐specific antigens, promoting individualized antitumor immune responses.
4. OMVs can be modified through genetic engineering or chemical methods to achieve multifunctionality. For instance, surface modifications with CaP^[^ ^126^ ^]^ or TA^[^ ^155^ ^]^ shells can shield the danger signals of OMVs, prolonging their circulation time in vivo. Additionally, OMVs can be conjugated with targeting peptides, cytokines, or ligands to precisely target tumor cells.^[^ ^96^ ^]^

Owing to the significant increase of potential in cancer immunotherapy, the modification of wild‐type OMVs is a crucial strategy and development direction. In conclusion, the engineering of OMVs has emerged as a versatile and promising strategy for advancing cancer immunotherapy. The intrinsic immunogenicity of OMVs, coupled with the ability to tailor their composition through genetic engineering, surface modifications, hybrid membrane fusion, and drug loading, presents an adaptable platform that holds significant potential for personalized and targeted cancer treatments. As we reflect on the current state of the field, it is evident that engineered OMVs have demonstrated promising results in preclinical studies and have even begun to make their mark in early‐phase clinical trials. The immunomodulatory capabilities of these vesicles, including their roles in antigen presentation and activation of immune responses, underscore their potential as effective cancer vaccines or adjuvants. Furthermore, the ability to incorporate tumor‐specific antigens and immunomodulatory molecules into OMVs allows for a customizable approach, addressing the unique challenges posed by different cancer types.

However, several challenges and opportunities lie ahead.

1. The translation of engineered OMVs from bench to bedside necessitates a deeper understanding of their safety profiles, long‐term effects, and potential side effects in diverse patient populations. Standardization of manufacturing processes and quality control measures will be crucial to ensure the reproducibility and scalability of these novel therapeutics. Future research directions should focus on refining engineering strategies to enhance the immunogenicity and specificity of OMVs, possibly through the identification of neoantigens or the incorporation of advanced genetic editing techniques.
2. Compared to adoptive cell therapies such as aAPCs, OMVs face challenges such as drug release, stability, and regulatory concerns. Unlike aAPCs, which can be engineered for precise release kinetics,^[^ ^175^ ^]^ OMVs may release the inner payload too quickly or too slowly, potentially reducing therapeutic efficacy. Meanwhile, OMVs can be less stable during long‐term storage and transport, whereas aAPCs are designed to be more robust.^[^ ^176^ ^]^ Most importantly, the usage of bacterial components in OMVs may raise additional regulatory and safety concerns, including the potential for unintended immune reactions or contamination, while aAPCs, being acellular and more defined, might encounter fewer regulatory hurdles. To achieve complementary advantages, investigations into the combination of engineered OMVs with other immunotherapeutic modalities could offer synergistic effects and improve overall treatment outcomes.
3. Besides, OMVs possess not only the benefits of a cell‐free system, nanoscale structure, stable loading capacity, and excellent biocompatibility, but also exhibit traits such as safety, facile modification, and straightforward industrialization. Thus, OMVs possess significant potential as carriers for delivering antitumor drugs such as reducing the cardiotoxicity of DOX,^[^ ^81^ ^]^ which improves its therapeutic efficacy. However, it is important to be cautious about certain limitations. Apart from drug‐release, OMVs often suffer from low drug‐loading capacity, which could potentially affect therapeutic efficacy without maintaining appropriate drug concentrations. Therefore, to advance the clinical applications of OMVs, several challenges need to be addressed including ensuring safety through rigorous evaluation after attenuation, optimizing structural properties for controlled drug release and enhanced drug loading, scaling up production to reduce costs, and navigating clinical ethical reviews.

In a broader context, exploring the potential of engineered OMVs in other disease contexts beyond cancer, such as infectious diseases or autoimmune disorders, could further broaden their therapeutic applications. Collaborative efforts between researchers, clinicians, and industry partners will be essential to overcome existing challenges, accelerate translational efforts, and bring engineered OMVs closer to mainstream clinical use.

In essence, the journey of engineered OMVs in cancer immunotherapy is at an exciting crossroad. With continued dedication to research, innovation, and collaborative endeavors, we envision a future where these adaptable vesicles play a pivotal role in revolutionizing cancer treatment, offering safer, more effective, and personalized therapeutic options for patients. The untapped potential of engineered OMVs invites a new era of possibilities in the dynamic landscape of immunotherapy, holding the promise of reshaping the way we combat cancer and other challenging diseases.

## Conflict of Interest

The authors declare no conflict of interest.

## Author Contributions

Z.L., and X.C. contributed equally to this work. Z. L., and X. C. conceived and drafted the manuscript, drew the figures and summarized the tables. B. F., D. F., X. L., R. X., T. L., S. W. discussed the concepts of the manuscript. F. C., and W. Z. approved the version to be submitted.
